# *Spirulina*-Templated Metal Microcoils with Controlled Helical Structures for THz Electromagnetic Responses

**DOI:** 10.1038/srep04919

**Published:** 2014-05-12

**Authors:** Kaori Kamata, Zhenzi Piao, Soichiro Suzuki, Takahiro Fujimori, Wataru Tajiri, Keiji Nagai, Tomokazu Iyoda, Atsushi Yamada, Toshiaki Hayakawa, Mitsuteru Ishiwara, Satoshi Horaguchi, Amha Belay, Takuo Tanaka, Keisuke Takano, Masanori Hangyo

**Affiliations:** 1 Iyoda Supra-Integrated Material Project, Exploratory Research for Advanced Technology (ERATO), Japan Science and Technology Agency (JST), 4259 Nagatsuta-Cho, Midori-Ku, Yokohama, Kanagawa 226-8503, Japan; 2 Research Society for Biotemplate Technology, 4259 Nagatsuta-Cho, Midori-Ku, Yokohama, Kanagawa 226-8503, Japan; 3 Division of Integrated Molecular Engineering, Chemical Resources Laboratory, Tokyo Institute of Technology, 4259 Nagatsuta-Cho, Midori-Ku, Yokohama, Kanagawa 226-8503, Japan; 4 Technology and Engineering Department, Materials Division, Sumitomo Metal Mining Co. Ltd., 5-11-3 Shimbashi, Minato-Ku, Tokyo 105-8716, Japan; 5 Advantest Laboratories Ltd., 48-2 Matsubara, Kami-Ayashi, Aoba-Ku, Sendai, Miyagi 989-3124, Japan; 6 DIC LIFETEC Co., Ltd., 12 Yawata-Kaigandori, Ichihara, Chiba 290-8585, Japan; 7 Earthrise Nutritionals, LLC., 113 E. Hoober Road Calipatria, CA 92233, USA; 8 Metamaterials Laboratory, Advanced Science Institute, RIKEN, Wako, Saitama 351-0198, Japan; 9 Institute of Laser Engineering, Osaka University, 2-6 Yamadaoka, Suita, Osaka 565-0871, Japan

## Abstract

Microstructures in nature are ultrafine and ordered in biological roles, which have attracted material scientists. *Spirulina* forms three-dimensional helical microstructure, one of remarkable features in nature beyond our current processing technology such as lithography in terms of mass-productivity and structural multiplicity. *Spirulina* varies its diameter, helical pitch, and/or length against growing environment. This unique helix is suggestive of a tiny electromagnetic coil, if composed of electro-conductive metal, which brought us main concept of this work. Here, we describe the biotemplating process onto *Spirulina* surface to fabricate metal microcoils. Structural parameters of the microcoil can be controlled by the cultivation conditions of *Spirulina* template and also purely one-handed microcoil can be fabricated. A microcoil dispersion sheet exhibited optically active response attributed to structural resonance in terahertz-wave region.

People have been fascinated by highly complex microstructures produced in nature^1^. Natural materials are sophisticatedly organized in response to specific functions, whose systematical organizing processes are superior to any engineering routes for smart materials in the light of multiplicity, optimal integration, and low energy consumption. In naturally occurring microstructures, important feature is their high dimensionality, which stimulates material scientists to design functional materials. Biomimetics or bioinspiration^2^,^3^,^4^, learning microstructures produced in nature, have drawn attention as one of breakthroughs on material science and processing. But profound understanding of formation mechanism established under natural selection process must be required to develop the biomimetic process. In fact, we have suggested collaborating with nature, *i.e*., biotemplating process, which directly borrows the natural microstructures for new material fabrications (Fig. 1). A concept of this process can lead to mass productivity with less energy. Main issue is how to control the natural microstructures. If we can find effective factors controlling the structures from various environmental conditions, the biotemplating process would have unlimited potential and will be global strategy for material development. We have focused three-dimensional helical feature in nature as biotemplate candidate^5^. Helical microalgae, *Spirulina* (*Arthrospira platensis*)^6^,^7^ naturally shaping left-handed (LH) open helical structure and already commercialized as nutritional supplements or food materials^8^, was employed in this study. Its helical structure is very sensitive against the environmental conditions, which should be strong advantage to exploit a variety of diameter, pitch, and handedness. Development of biotemplating process using *Spirulina* can achieve mass production of microcoil (μcoil), which is currently manufactured by precision machining or lithography one by one. Since Chen et al. first coated the *Spirulina* surface with magnetic ferrite^9^, several biotemplating processes using the *Spirulina* have been reported in the field of material science. However, no function specific to its helical microstructure has yet been discovered.

## Results

### Structure-controllability of *Spirulina* biotemplate

**Figure 2:**
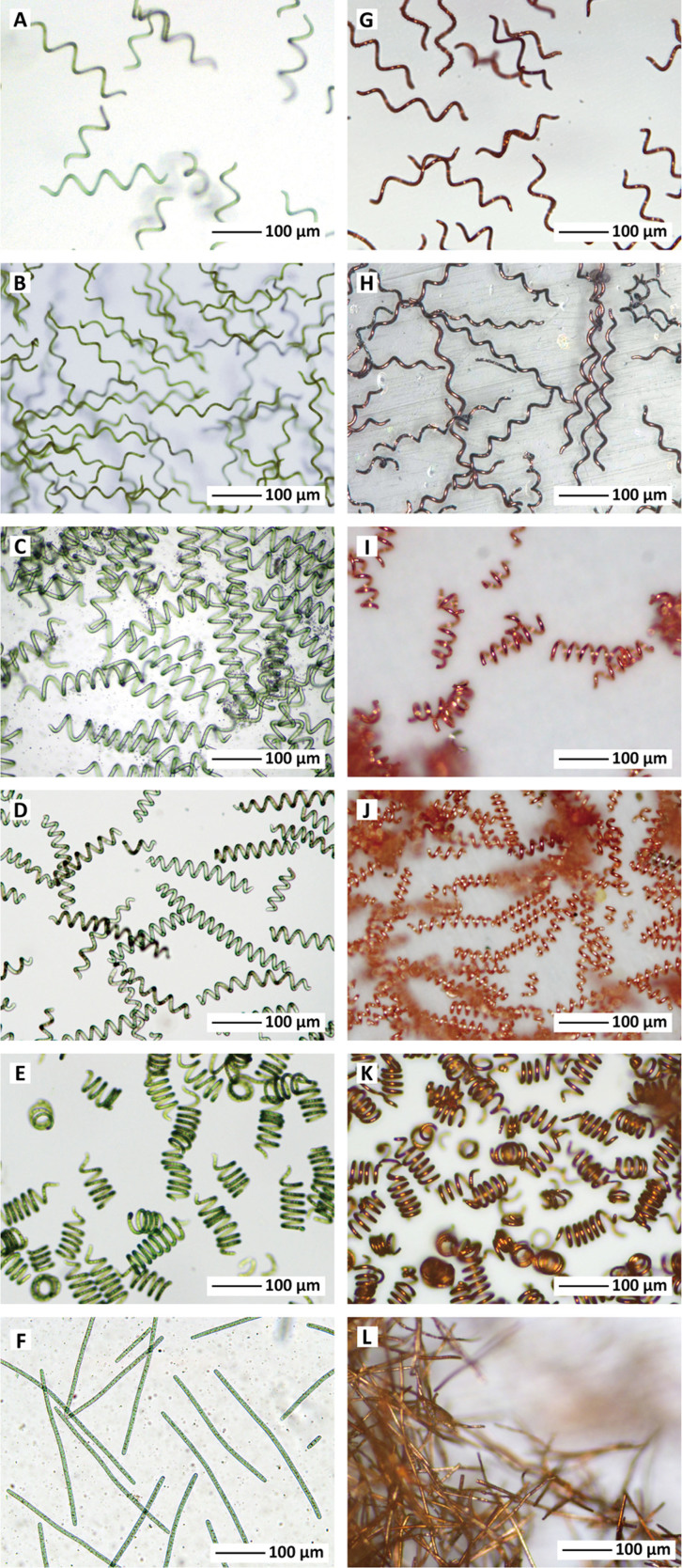
Left-handed (LH) *Spirulina*-templated microstructures. The *Spirulina* basically forms LH helix but its structural parameters such as helical pitch, length, number of turns, and handedness are of variety based on a kind of strains. Sensitive strain, NIES-46, gradually tightens the helical pitch as the cultivation proceeded with stronger light intensity and higher temperature. The systematic adjustment of the helical pitch, *L_free_*/*N*, can be demonstrated; (a)-(e), LH template-1 to -5. (f) Linear strain was prepared by the pure cultivation of laboratory-derived single trichome. The biotemplating process was successfully carried out to generate the copper μcoils whose structures are followed by those of LH templates: (coil number, *L_free_*/*N*); (g) LH μcoil-1, 77 μm; (h) LH μcoil-2, 55 μm; (i) LH μcoil-3, 27 μm; (j) LH μcoil-4, 20 μm; (k) LH μcoil-5, 16 μm. (l) The straight copper wire was also properly templated from the linear strain.

The LH helix of *Spirulina* is a common structure found in nature and easily obtained as the stock strain from culture collections. Under the conventional cultivation condition^10^, average feature size of the LH *Spirulina* (Fig. 2a) were 6 μm in wire diameter (*d*), 43 μm in coil diameter (*D*), 174 μm in free length of coil (*L_free_*), 74 μm in coil pitch (*L_free_/N*), 2.4 in turn number (*N*), and 29 ° in pitch angle (*α*) (for graphical illustration of the symbols, see Fig. S1). The number of *Spirulina* in 1 mL increased from 10^2^ to 10^5^ within one week (Methods and Fig. S2). During the cultivation, the *d*, *D*, and *L_free_/N* remained with narrow size distributions (around 5–15% of relative standard deviation, RSD), while the *L_free_* had a higher RSD around 20% because *Spirulina* grows along the long axis direction (Fig. S3). It has been known that the *Spirulina* forms various helical features and even linear shape under different environments. In this study, the *L_free_/N* was systematically controlled by varying cultivation temperature and light intensity (Methods and SI-II)^11^. The series of LH *Spirulina* with five different helical features in Fig. 2 are numbered as LH template-1 to -5. Linear *Spirulina* often found in laboratory cultivation of regular helical strains was obtained in pure culture (Fig. 2f). Such flexible morphologies against the environment can achieve distinction as microstructured-materials separately from commonly-used genetic control.

### *Spirulina*-based biotemplating process

We have designed the biotemplating process using electroless plating technique, which can generate smooth metal layer selectively on surface of targeting object to be plated. In this study, the process includes (i) fixation of *Spirulina*, (ii) Pd catalyzation (Pd nanoparticle adsorption as plating catalyst nuclei^12^,^13^), and (iii) copper electroless plating (Methods, see also Supplementary SI-III). The process (i) employed general method of tissue fixation by glutaraldehyde, which cross-links amine groups of amino-acid side chains in proteins^14^. The process (ii) includes two steps with Pd ion adsorption and reduction of the Pd ion to form the metallic Pd, which can work as catalyst oxidizing reducing reagent for the metal deposition from the plating bath. For the electroless plating, bath load, *i.e.*, the total surface area of *Spirulina* to amount of plating bath, was adjusted to be 200 cm^2^/L. In the laboratory scale with 1 L of plating bath, one batch from 20 mL of the *Spirulina* cultivation medium with 10^5^ mL^−1^ in *Spirulina* concentration gave approximately 2 million μcoils (90 mg, 80% yield). The resulting μcoils were quantitatively and qualitatively characterized by ICP, XPS, XRD, and EDX-SEM at different stage of biotemplating process (for the detailed information, see supplementary SI-III). It was found that the Pd catalyst was adsorbed to the surface as well as inside the tissue of *Spirulina* with almost 2 vol% in volume fraction to the *Spirulina*. The μcoil contained the metallic Pd of >80% and a little amount of PdO_x_ in the nanoparticles.

The copper layer deposited on the *Spirulina* was consisting of the metal (>90% in content) with the oxides at the surface. The μcoils looked reddish brown with metallic luster and had tubular structure with around 550 nm in thickness, whose interior space still included the fixed *Spirulina* template (Fig. S15). The thickness can be controlled by changing the plating time and also the bath load, here, we demonstrated with 200 cm^2^/L in the bath load (20-mL loading amount) to obtain the thickness enough to exhibit bulk-like electric property. The efficiency of Cu deposition and relationship between the bath load and the thickness were discussed in supplementary SI-IV. The optical micrographs of μcoils (LH μcoil-1 to -5) fabricated from corresponding LH template-1 to -5 are shown in Figure 2g-2k along with straight copper wire (Fig. 2l) from the linear strain. Emphasis should be placed on that the microstructures after the biotemplating processes were faithfully transferred from those of the *Spirulina* templates, for example, in case of LH μcoil-1, *d* = 7.0 μm (15%RSD), *D* = 41 μm (14%), *L_free_* = 174 μm (26%), *L_free_*/*N* = 77 μm (9%), *N* = 2.3, and *α* = 31 ° (see also Fig. S16).

**Figure 3:**
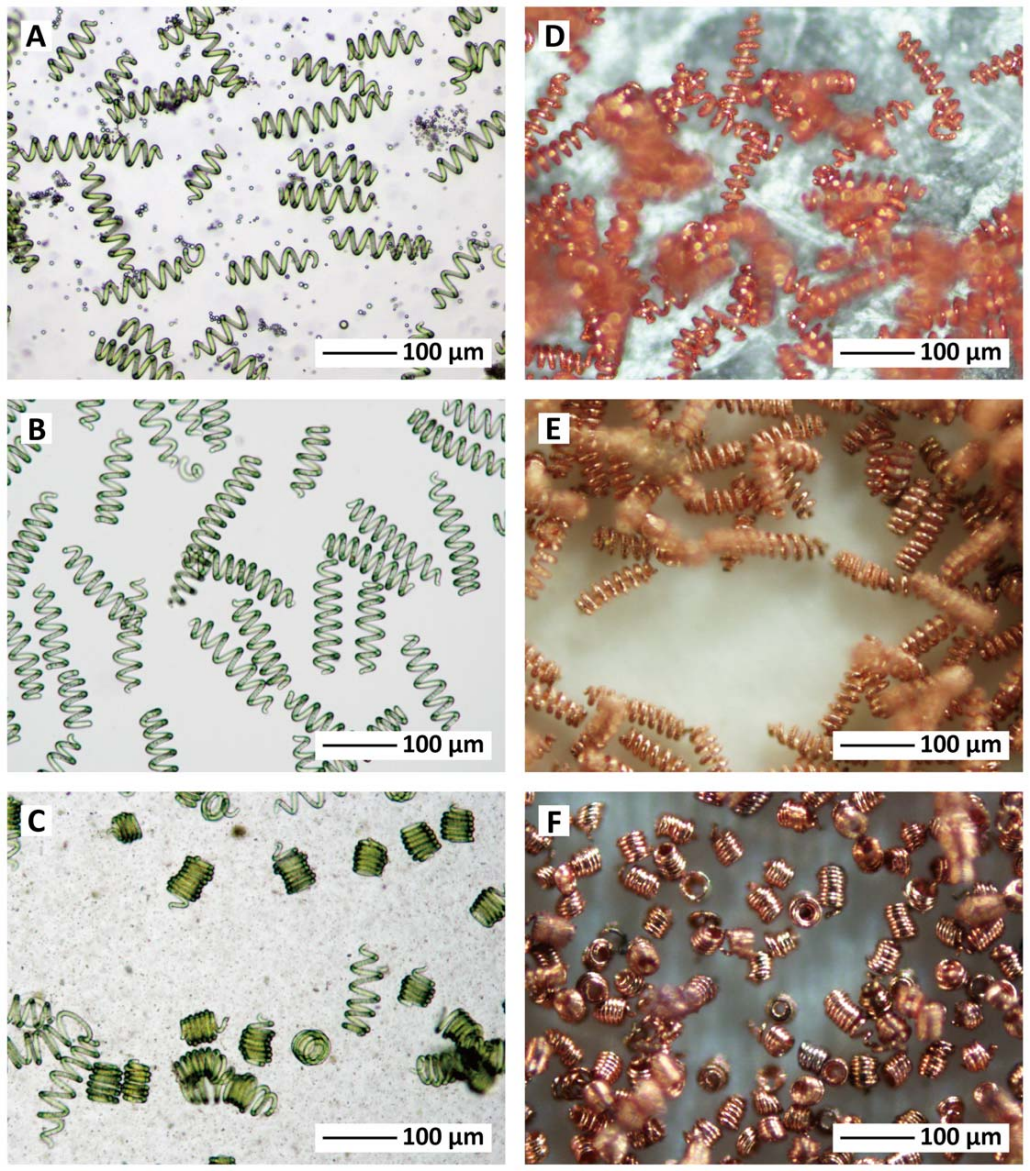
Right-handed (RH) *Spirulina* and their biotemplated products. RH template-1 to -3 with three different *L_free_*/*N*s, (a) – (c), generated the corresponding RH μcoils: (coil number, *L_free_*/*N*); (d) RH μcoil-1, 19 μm; (e) RH μcoil-2, 14 μm; (f) RH μcoil-3, 6 μm.

Right-handed (RH) strains of *Spirulina* are rare but they are found both in nature and in culture. Reversal of helical handedness from left to right and vice versa has been observed and ascribed to genetic drift or environmental factors like temperature uplift or mechanical stress^15^. One such RH strain obtained from Earthrise Nutritionals, California, USA, was grown in pure culture from a single trichome. The RH *Spirulina* was more tightly coiled smaller than around 20 μm in *L_free_*/*N*. The RH templates and the corresponding μcoils were prepared in the same manner as the LH series (Fig. 3 and Fig. S17). The structural parameters for all of μcoils and referenced samples are summarized in Table 1.

### Can the μcoil behave as chiral electromagnetic material?

**Figure 4:**
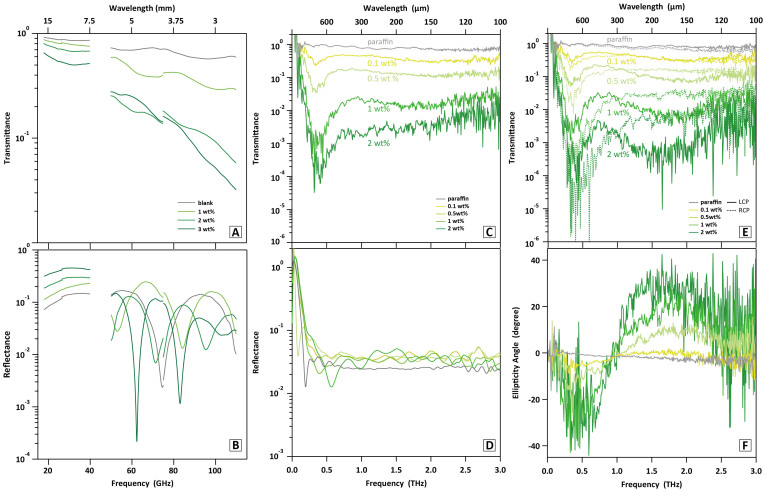
Electromagnetic response of copper μcoil sheets. (a), (b) Transmission and reflection spectra in the region of 18–40 GHz and 50–110 GHz with a prescribed amount of μcoils in paraffin and silicon matrix, respectively. (c), (d) THz transmission and reflection spectra of LH μcoil-1 using non-polarization mode. The concentration dependence was evaluated with four different wt% (0.1, 0.5, 1, and 2 wt%, colored with pale to dark green). (e), (f) THz transmission spectra under circular polarization mode and their ellipticity angle spectra. Those of a μcoil-free paraffin sheet (gray line) are also depicted as reference. LCP (solid line) and RCP (dotted line) show transmittances against left-handed and right-handed circular polarizations, respectively.

We prepared dispersion silicon sheets of LH μcoil-1 for transmission and reflection spectroscopies in the region of millimeter wave with free space method (Fig. 4a and 4b, SI-VI). The logarithmic transmittance decreased as the concentration of μcoil was increased. Reflection components were almost constant against the concentration change in the region of V-band and W-band (50 to 110 GHz), although multi-reflections based on the sheet thickness were observed in every sample. The sheet showed remarkable transmission loss possibly based on absorption, for example, less than 10% transmittance at 60 GHz in case of 3 wt%. A monotonous decrease in transmittance at higher frequency region led us to realize resonance specific to the μcoil structure in the region of THz wave. THz time-domain spectroscopy (THz-TDS) with non-polarization mode was measured with the LH μcoil-1 dispersed into paraffin (Fig. 4c and 4d). No spectral feature with negligible reflection loss was observed in the μcoil-free paraffin sheet. Considerable transmission loss was observed over the entire region from 0.2 to 3.0 THz for the paraffin sheets containing only small amount (~2 wt%) of the LH μcoil. The μcoil concentration showed linear relationship to optical density converted from the transmittance, which is consistent with Lambert-Beer law (Fig. S19). The concentration dependence proved no anomalous radiation from imperfect dispersibility of μcoils. Furthermore, reflectance of the sheet was around 3%, so that the transmission loss was attributed to absorption of THz wave (for the spectra of all the LH μcoils with non-polarization mode, see Fig. S20). In order to evaluate optical activity of μcoil, THz-TDS combined with polarimetric analysis (THz-TDS-PA, SI-VII)^16^,^17^ was conducted with the same sample, LH μcoil-1. Significant difference was found in transmittances against RH and LH circular polarizations (Fig 4e). The spectrum of ellipticity angle gave negative and positive bands in around 0.5 and 1.5 THz, respectively (Fig. 4f). The spectroscopic evidences supported that the μcoil-dispersion sheet exhibits optically active THz response. Here, we considered function of the structure handedness of LH μcoil for the optical activity found in the isotropic dispersion, unlike metal helix array^18^,^19^. Chigrin et al.^20^ reported that achiral microparts can even effectively exhibit elliptical dichroism only based on their twisted configuration.

### The LH and RH μcoils are optically active isomers

**Figure 5:**
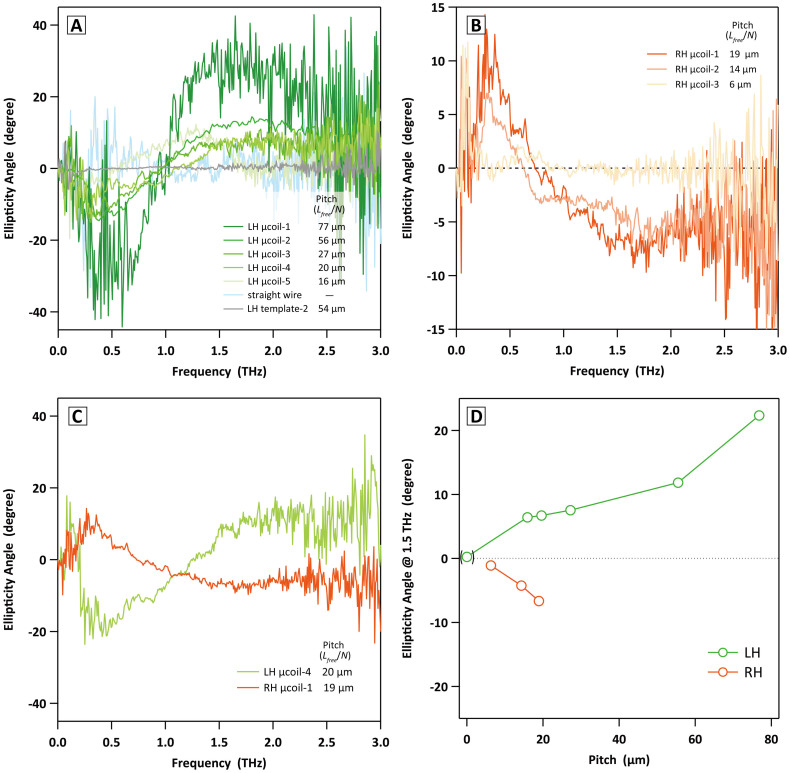
Dependence of helical shape and handedness of μcoil on optically active response. (a), (b) Ellipticity angle spectra of μcoil-dispersion paraffin sheets were summarized into series of LH μcoils and RH μcoils as well as reference samples. All of samples contained 2 wt% in the paraffin matrices. The coil numbers and their *L_free_*/*N* values are depicted in insets. The RH μcoil shows laevorotation with ellipticity angle of opposite sign against the LH case. The intensity of ellipticity angle decreased and also the inversion frequency, where occurs the sign inversion from negative to positive for LH μcoil and opposite inversion for RH μcoil, shifted toward lower frequency region as the *L_free_*/*N* became smaller. Dispersion sheets of straight copper wire and freeze-dried LH template-2 with 54 μm in *L_free_*/*N* showed no spectral features, which ensures the optical activity specific to the metal helical microstructure. (c) Ellipticity angle spectra of LH μcoil-4 and RH μcoil-1 selected as enantiomeric pair. (d) Ellipticity angle at 1.5 THz as a function of *L_free_*/*N*. The value from the straight copper wire was depicted with parenthesis at 0 μm in pitch.

The variety of μcoils fabricated through systematic control of helical structures of *Spirulina* biotemplates enables us to experimentally evaluate structure-specific chiral electromagnetic responses. As references, it was confirmed that the dispersion sheets containing the straight copper wire and freeze-dried LH template-2 showed optical inactive. All of LH μcoils gave the similar spectral features of their ellipticities as the LH μcoil-1, *i.e*., negative sign at 0.5 THz and positive one above 1.0 THz (Fig. 5a). At the same peak frequencies, the RH μcoils exhibited opposite signs of ellipticities (Fig. 5b). The LH μcoil-4 and RH μcoil-1 were selected here as enantiomeric pair with similar *L_free_*/*N* values. The ellipticities of the enantiomeric pair obviously showed mirror-image spectra with opposite signs; +15° for LH μcoil-4, -10° for RH μcoil-1 at 2.0 THz (Fig. 5c). Separately, another sheet containing a racemic mixture of LH and RH μcoils with 1 wt% each traced the average of individual ellipticity angle spectrum, meaning no peaks with flat spectral feature (Fig. S21). The frequency showing sign inversion in ellipticity angle spectrum resulted in peak of rotation angle (Fig. S22). Therefore, it came to light that the LH μcoil emits the RH elliptical polarization and vice versa above the sign inversion frequency. That is to say, the LH μcoil shows dextrorotation and the RH one does levorotation.

## Discussion

There are three points of view to discuss the THz response observed in this study; the functional frequency region for μcoil response, the frequency at 0 ° of ellipticity angle (sign inversion frequency), the degree of ellipticity angle. First, the peak of ellipticity angle at higher frequency showed a tendency to shift inversely proportionally to length of wire to make one pitch, *L_wire_*/*N*. The relationship between the *L_wire_*/*N* and the functional frequency has been well explained by helical antenna theory^21^ and helix array system^22^, whose resonant wavelength is defined as *L_wire_*/*N* < *λ* < 2 *L_wire_*/*N*. Briefly, it can be said that the μcoil sheet operates in a half-wave resonance mode (*λ = * 2 *L_wire_*/*N*) (Table 1 and SI-VIII). In the lower frequency region, the correlation specific to the structure was not observed. The possible reason is that there are many other resonance modes such as dipole mode, internal resonance based on *L_free_*/*N*, and inter-coil coupling. Second, the sign inversion frequency shifted toward lower frequency as the sample was varied from loose to tight μcoils, which appeared closely related to the *α*. The sign inversion shift observed here was found as having dominant self-resonant frequency, *f_0_*, expressed with inductance, *L*, and capacitance, *C*, *i.e.*, 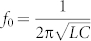. Since the tight μcoil has the shorter *L_free_*/*N* as well as the larger *N* and/or *D*, the *LC* value should be larger, consequently giving the lower *f_0_*. Finally, as a crucial sense, the intensities of ellipticities decreased as the *L_free_*/*N* became smaller, in common with both of LH and RH μcoils (Fig. 5d). It has been reported that the helix array theoretically shows the same phenomenon^22^. In an extreme case, a ring or wire shape having *L_free_*/*N* = 0 or *α* = 90 °, respectively, loses chirality. We are also considering a similarity between the THz chiral behavior of μcoil and exciton chirality of optically active molecule. Similar to Davydov splitting in molecular crystal, an interactive pair of dipole moments induced by electric or magnetic field change along the helix causes the resonance energy split into two. Due to their twisted configuration, both resonances are allowed and act asymmetrically electromagnetic induction, resulting in the ellipticities with opposite signs. Further discussion on the optical activity with helical antenna response is under investigation. The alignment control in the sheet may allow us to fully understand the THz responses in our system.

The biotemplating process was successfully demonstrated to develop a new class of 3D-structured material. The *Spirulina*-based μcoil exhibited the optical activity, of which the conditions such as the sense of wave rotation and the operation frequency were controlled by the structural parameters of μcoils. A wide distribution on the structural parameters, always attendant on the biotemplating process, would be rather effective for broadband operation. The present fabrication process can be applied for various distinctive biological tissues and microorganism with mass-productivity and a wide variety of material form, which will promise new strategy for material design.

## Methods

### *Spirulina* cultivation and shape control

The LH *Spirulina* (NIES-39 and -46, stock strains in National Institute for Environmental Studies) was cultivated with conventional aqueous media^10^ in an open-air water tank at room temperature under fluorescent light (2,500 lx). For the shape control of *Spirulina* template, the cultivation temperature and light intensity were raised up to 35 °C and 7,500 lx, respectively. The LH template-1 was obtained from the standard condition and the LH template-2 to -5 were collected every fourth day from the mass cultivation medium with the controlled condition under way into the tightening of helix, decreasing of *L_free_/N*. The linear strain was prepared by pure cultivation of the single trichome occurred by longer cultivation at the constant condition for more than 2 months.

### Biotemplate process

The *Spirulina* was collected by a nylon mesh filter with 355 mesh and resuspended into a 100 mL of phosphate buffer solution (pH = 7.0) including 4% glutaraldehyde as tissue fixation solution. The optical density of the *Spirulina* suspension was adjusted to be around 2.0 at 550 nm with an optical pass length of 1 cm, which closely equals to the order of 10^5^ in the number of *Spirulina* per 1 mL. The suspensions were left in ambient atmosphere overnight to complete fixation of *Spirulina* tissues. For further storage, they are kept in cool dark place so that the *Spirulina* can preserve their shapes and be used as the biotemplates for several years. Copper electroless plating onto the surface of *Spirulina* was conducted by the use of commercial products from Okuno Chemical Industries Co., Ltd.: for delipidation treatment, OPC-370 Condiclean MA; for Pd catalyzation (adsorption of Pd chloride based alkaline ionic catalyst), OPC-50 Inducer; for activation of the catalyst (reduction of Pd ion by dimethylamine borane), OPC-150 Cryster MU; for copper electroless plating, OIC Copper. All kinds of plating baths were initially prepared for 1 L. The preparation of plating baths and each processing time were listed in Table S1. A 20 mL of fixed *Spirulina* suspension was filtered with the nylon mesh to collect the number of *Spirulina* with 2 × 10^6^, corresponding to 12 mg in weight of dried *Spirulina* and 200 cm^2^/L in bath load. It is noted that the surface area of one *Spirulina* is typically around 1 × 10^4^ cm^2^. The *Spirulina* collected on the filter was immediately added to the baths before started to dry and stirred by mechanical stirrer with 300 rpm. The filtration and rinsing with water were successively carried out to switch the bath. After the final step with OIC Copper, the resulting copper μcoils were washed well on the filter and dispersed into 100 mL of distilled water overnight. The μcoils were collected by vacuum filtration using membrane filter (0.8-μm pore size, ATTP type, Isopore™ Track-Etched Membrane Filters) and dried in the atmosphere. The amount of μcoil generally averaged 90 mg, which gave 80% yield in the case of μcoil having 550 nm in the thickness of copper layer.

## Author Contributions

K.K., S.S., A.Y. and T.I. started this project; T.F. and W.T. contributed to the design of experiment; Z.P., M.I., S.H. and A.B. conducted *Spirulina* cultivation; K.T. and M.H. performed the THz-TDS and data analysis; T.I. and M.H. coordinated the study with contributions from T.H., T.T. and K.N.; K.K. wrote the manuscript.

## Supplementary Material

Supplementary Information Supplementary Information

## Figures and Tables

**Figure 1:**
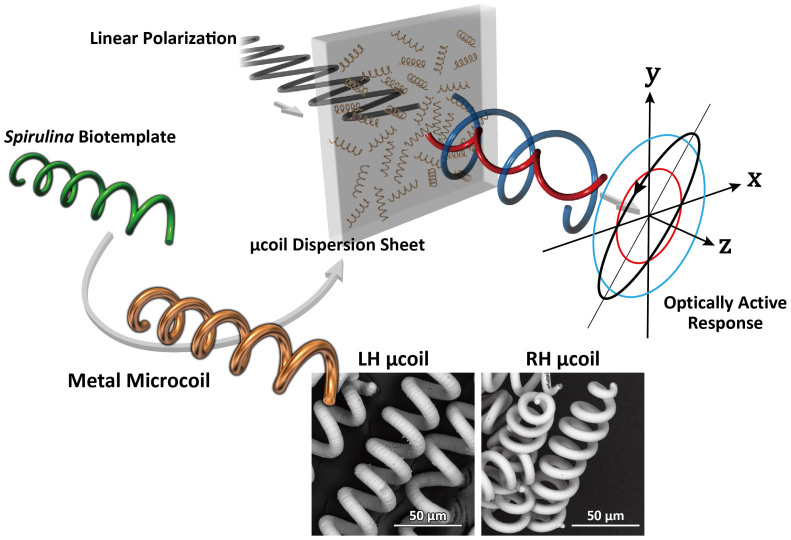
Fabrication of *Spirulina*-templated microcoil (μcoil) and its optical activity against THz wave. The *Spirulina* (*Arthrospira* *platensis*) was utilized as biotemplate to mass fabricate 3D helical μcoils. An enantiomeric pair of left-handed (LH) and right-handed (RH) μcoils can be fabricated with 100% of optical purities as SEM images show. The THz time-domain spectroscopies with polarimetric analyses (THz-TDS-PA) of paraffin sheets containing the μcoils were measured to examine optical activities and structural resonances based on the helical structures.

**Table 1:** The geometric parameters of μcoil

Parameters	wire diameter	coil diameter	free lengthof pitch	number of turn	free lengthof coil	length of wire for one pitch	length of wire for one coil	pitch angle^1^	theoretical frequency region^2^	detected frequency region^3^
symbol	*d*	*D*	*L_free_*/*N*	*N*	*L_free_*	*L_wire_*/*N*	*L_wire_*	*α*	*F_d_*	*F_0_*
Units	μm	μm	μm	-	μm	μm	μm	degree	THz	THz
**LH μcoil-1**	7	41	77	2.3	174	150	339	30.9	0.67–1.33	0.5–1.5
**LH μcoil-2**	7	26	56	4.5	248	99	441	34.0	1.02–2.03	0.5–1.6
**LH μcoil-3**	9	35	27	5.7	153	113	642	13.8	0.88–1.77	0.5–1.9
**LH μcoil-4**	7	22	20	5.6	111	72	399	16.1	1.39–2.78	0.5–2.1
**LH μcoil-5**	8	46	16	5.1	81	145	736	6.3	0.69–1.38	0.4–1.2
**RH μcoil-1**	8	30	19	6.8	130	96	658	11.4	1.04–2.08	0.4–1.6
**RH μcoil-2**	8	30	14	7.9	110	95	749	8.5	1.05–2.10	0.3–2.0
**RH μcoil-3**	6	33	6	7.0	44	104	725	3.5	0.96–1.93	*n/a*
**Straight wire**	6	*n/a*	*n/a*	*n/a*	320	*n/a*	320	90	*n/a*	*n/a*
**Freeze-dried *Spirulina***	5	20	44	4.3	188	77	328	35	1.30–2.61	*n/a*

^1^The pitch angle equals to tan^−1^(*L_free_*/*N*)/(π*D*).

^2^The frequency region means that the μcoil emits elliptical polarization with the opposite handedness within the range and can be predicted with *L_wire_*/*N* < *λ_0_* < 2*L_wire_*/*N*, as defined in helical antenna array. The wave propagates in paraffin matrix (*n* = 1.5), so that the frequency is given by 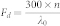.

^3^The frequency region was defined as the difference between two peaks of ellipticity angles.
