# Supplementary material for: Spirulina-Templated Metal Microcoils with Controlled Helical Structures for THz Electromagnetic Responses
Source: Sci Rep. 2014 May 12;4:4919. doi: 10.1038/srep04919 (PMC4017220; doi:10.1038/srep04919)
Supplement: Supplementary Information [file srep04919-s1.pdf]

Supplementary Information

Spirulina-Templated Metal Microcoils with Controlled Helical Structures for THz Electromagnetic Responses

Kaori Kamata, Zhenzi Piao, Soichiro Suzuki, Takahiro Fujimori, Wataru Tajiri, Keiji Nagai, Tomokazu Iyoda, Atsushi Yamada, Toshiaki Hayakawa, Mitsuteru Ishiwara, Satoshi Horaguchi, Amha Belay, Takuo Tanaka, Keisuke Takano, & Masanori Hangyo

Sections

SI-I. Symbols for the structural parameters of microcoil

SI-II. Culture condition and size distribution of *Spirulina*

SI-III. *Spirulina*-based biotemplating process

SI-IV. Control of copper layer thickness

SI-V. Structural parameters of copper μcoils

SI-VI. μCoil-dispersed sheet for evaluation of electromagnetic response

SI-VII. THz-TDS-PA setup

SI-VIII. Geometric parameters of samples and summary

SI-I. Symbols for the structural parameters of microcoil (μcoil)

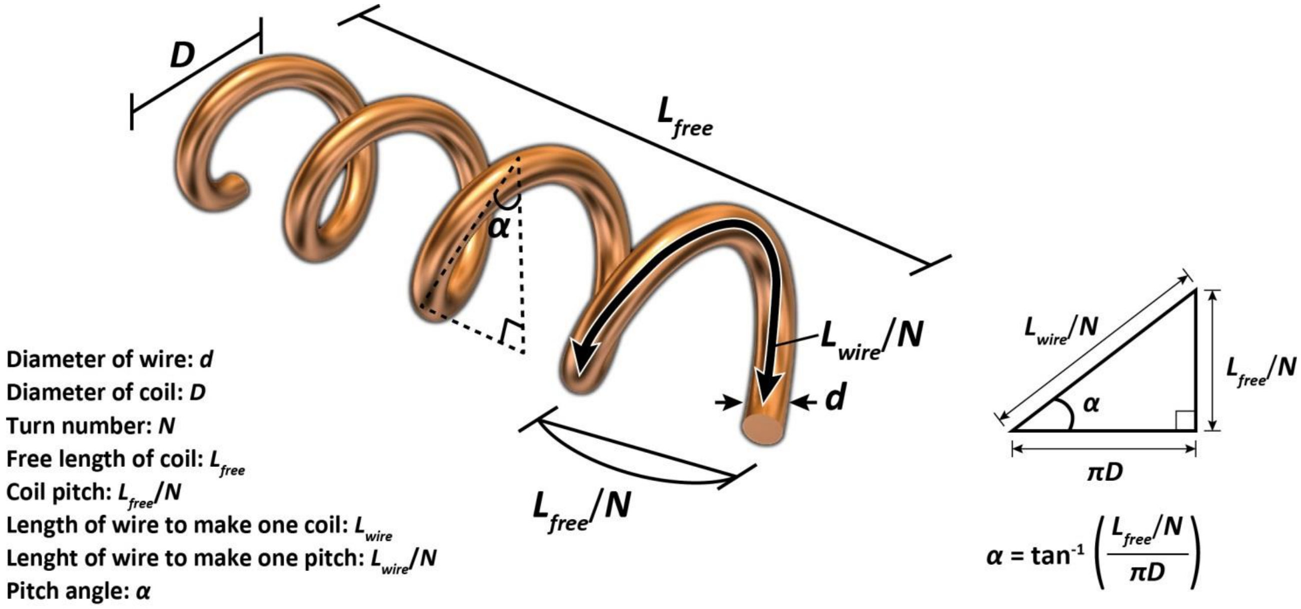

Figure S1 Symbols for the structural parameters of μcoil.

SI-II. Culture condition and structure observation of *Spirulina*

Spirulina, a blue-green algae in natural helical shape, were obtained by division from National Institute for Environmental Studies. The strain we used for the standard biotemplate was NIES-39, originally came from Lake Chad in Central African Republic, while for control of helical pitch, we selected NIES-46, from Lake Texcoco, due to its controllability on the helical structure under the different conditions of cultivation. Both of the strains have left-handed (LH) helix structure. The *Spirulina* was propagated in SOT (*Spirulina* Ogawa-Terui) medium at different environmental factors, temperature and light intensity. It has been known that physical and chemical conditions may affect the helix geometry (ref. 11 in the main text). At room temperature under fluorescent light irradiation with 2500 lx in our lab., the number of *Spirulina* doubles in a day, so that we are doing the routine cultivation from 10^2 to 10^5 in the *Spirulina* concentration (mL^{-1}) for almost one week. The growth curve was well fitted with an exponential function, *i.e.*, $y = N_0 \exp(ax)$, where the N_0 is the initial concentration of *Spirulina* and a is coefficient (Fig. S2). By optical microscopy (Olympus, BX51), the wire diameter (d), diameter of helix (D), pitch (L_{free}/N), turn number (N), free length (L_{free}), and pitch angle (α) of *Spirulina* (NIES-46) are found as approximately 6 μm, 43 μm, 74 μm, 2.4, 174 μm,

and 29 °, respectively (Fig. S3A-D). The culture temperature and light intensity were raised to 35 °C and 7500 lx, respectively, but the other factors were fixed. After the cell growth became stationary, the structural parameters were observed. It was found that the culture at 35 °C under the stronger intensity of light produced tight helix with L_{free}/N = 16 μm drastically smaller than the case at room temperature. In order to obtain the intermediate L_{free}/N between 77 and 16 μm, sampling approach was carried out by collecting *Spirulina* from the mass cultivation medium before the helix shape became stationary. Here, five different LH *Spirulina* having 77 μm, 55 μm, 27 μm, 20 μm, 16 μm in L_{free}/N were prepared as biotemplates, which were numbered as LH template-1 to -5. The LH template-2 was originated from the strain of NIES-39, the others were from the same strain as NIES-46.

Such flexible morphologies in the *Spirulina* offer many advantages on the fabrication of μcoils, which could show wide range of electromagnetic applications based on their controlled structural parameters.

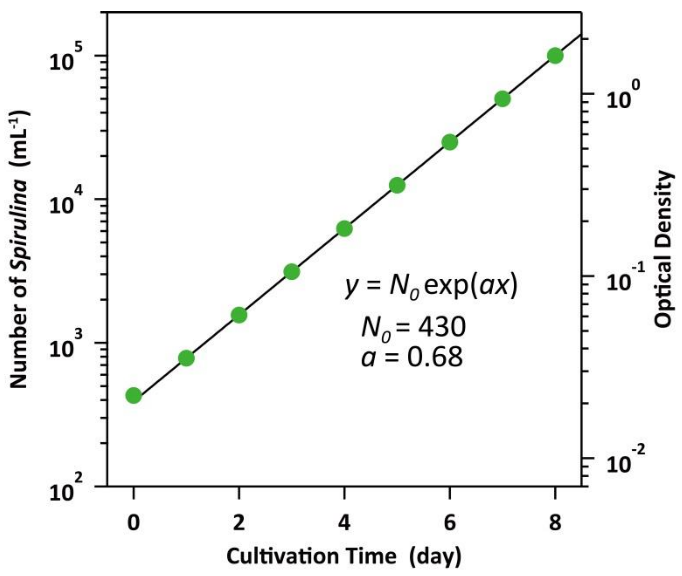

Figure S2 Growth curve of *Spirulina* under the regular cultivation condition. The number of *Spirulina* (dot) in 1 mL of cultivation medium was plotted as a function of cultivation time in logarithmic scale. Initial *Spirulina* concentration was 430 mL^{-1}. For the data collections, the number of *Spirulina* in 10 μL was counted under the optical microscope after dilution of the *Spirulina* suspension with water by 100 times. The growth curve well follows the exponential function (solid line). Experimentally, we monitored the optical density to estimate the *Spirulina* concentration.

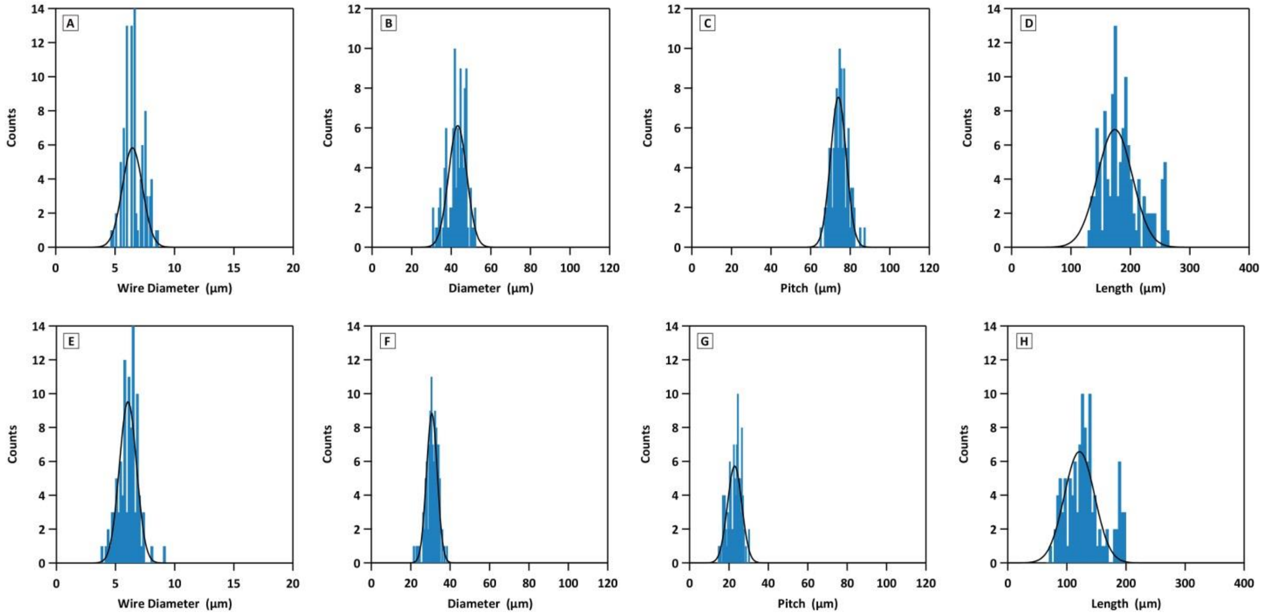

Figure S3 Histograms of structural parameters on helix geometries of *Spirulina*. (A), (E) Wire diameter (d), (B), (F) coil diameter (D), (C), (G) coil pitch (L_{free}/N), and (D), (H) free length of coil (L_{free}) were evaluated for the *Spirulina* LH template-1 and RH template-1, cultivated at room temperature with 2000 lx in light intensity. The black lines indicate Gaussian distributions to have averaged values. The structural parameters and percent relative standard deviations (%RSD) for the LH template-1 (A-D) are found as; d = 6 μm (13 %), D = 43 μm (11 %), L_{free}/N = 74 μm (5 %), L_{free} = 174 μm (17 %), for the RH template-1 (E-H), d = 6.1 μm (12 %), D = 31 μm (9 %), L_{free}/N = 23 μm (16 %), N = 7, and L_{free} = 121 μm (21 %).

We have failed to find RH *Spirulina*, although various cultivation conditions were tried. As mentioned in the main text, to search the naturally alive RH *Spirulina* became the possible candidate. A strain of RH *Spirulina* was obtained from Earthrise Nutritionals, LLC, a wholly owned subsidiary of DIC Corporation. One piece of RH *Spirulina* was picked up and transferred to cultivation medium for the pure cultivation. It took almost one month to have 1 L of cultivation medium with almost 10^5 mL^{-1} in concentration of RH *Spirulina*. The structural parameters of typical RH *Spirulina* (RH template-1) and their %RSDs were observed as d = 6.1 μm (12 %), D = 31 μm (9 %), L_{free}/N = 23 μm (16 %), N = 7, and L_{free} = 121 μm (21 %) (Fig. S3E-H). The L_{free}/N of RH *Spirulina* was controlled in a similar manner to the way of LH case for the preparation of RH template-1 to -3.

SI-III. *Spirulina*-based biotemplating process

Table S1 Procedure on Cu μcoil fabrication via electroless plating.

Process	Reagent	concentration	temperature	treatment time
**(i) Fixation**	glutaraldehyde	4 %	r.t.	over night
**(ii) Pd catalyzation**				
**Delipidation**	OPC-370 Condiclean MA	50 ml/L	40 °C	5 min
filtration and washing				
**Pd catalyzation**	OPC-50 Inducer A (30 mmol/L Pd ion)<br>OPC-50 Inducer C	(pH 12)<br>25 ml/L<br>25 ml/L	40 °C	5 min
filtration and washing				
**Activation** (reduction of Pd ion)	OPC-150 Cryster MU	75 ml/L	25 °C	5 min
filtration and washing				
**(iii) Cu electroless plating**	OIC Copper 1 (0.6 mol/L Cu ion)<br>OIC Copper 2<br>OIC Copper 4	20 ml/L<br>9 ml/L<br>100 ml/L	40 °C	10 min
-filtration and washing<br>-stored in water overnight<br>-filtration and drying				
**Cu μcoil**				

Each electroless plating bath was prepared for 1 L including prescribed amount of each plating reagent listed in Table S1. It is noted that the bath for Pd catalyzation and Cu electroless plating were prepared to include Pd ion with 0.8 mmol/L and Cu ion with 20 mmol/L, respectively. The amount of fixed *Spirulina* suspension in phosphate buffer solution with 4 % glutaraldehyde can be varied up to 40 mL, meaning that the total surface area of *Spirulina* against the volume of plating bath is limited to 400 cm^2/L. Higher total surface area resulted in strong entanglement and aggregation and also imperfect Cu coating on the *Spirulina* surface.

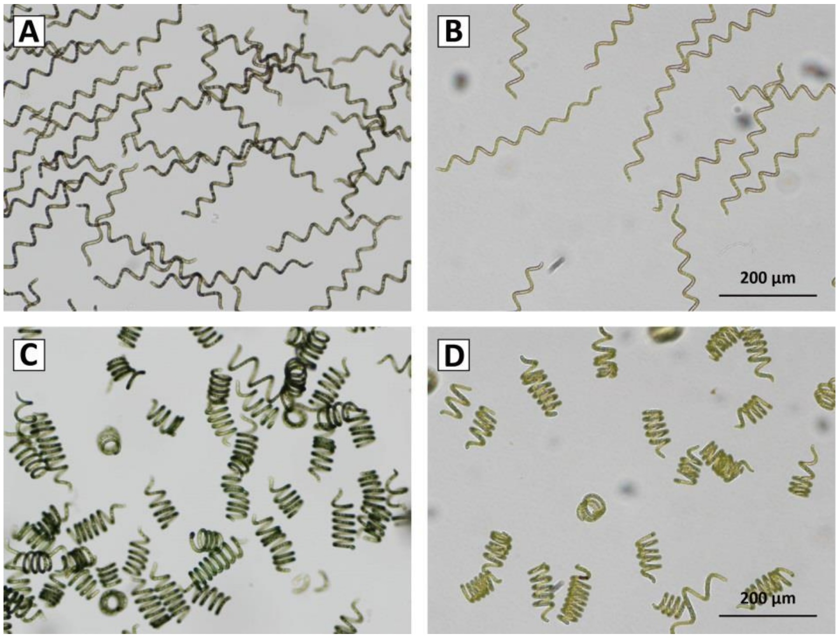

Figure S4 Optical micrographs of LH template-2 (A, B) and LH template-5 (C, D) at different stage of biotemplating process; A and C, after the fixation with glutaraldehyde; B and D, after the activation process reducing Pd ion adsorbed to *Spirulina*.

Characterization of Pd catalyst nuclei adsorbed to *Spirulina*.

The shape preservation of *Spirulina* templates was ensured after fixation with glutaraldehyde and also after pretreatment, the activation process of Pd catalyst (Fig. S4). The Pd-based catalyst nuclei adsorbed to the *Spirulina* indicated in Fig. S4B were characterized by X-ray photoelectron spectroscopy (XPS, Shimadzu, ESCA-3400) as forming metallic Pd with the content of 86 % and a little amount of oxides (PdO_x) (Fig. S5). The experimental curve in the Pd 3*d* region was fitted with Gauss function at the already-identified Pd $3d_{5/2}$ and Pd $3d_{3/2}$ peak positions[1]. The Pd 3*d* doublet appeared at 340.7 and 335.4 eV was reasonably assigned to metallic Pd with the expected intensity ratio of 1.5 and the other doublet at 338.0 and 343.3 eV was assigned to electron-deficient Pd normally described as PdO_x. The intensity ratio of peaks for metallic Pd and PdO_x in the Pd $3d_{5/2}$ range was 6.15, leading to that 86 % of Pd content was metallic.

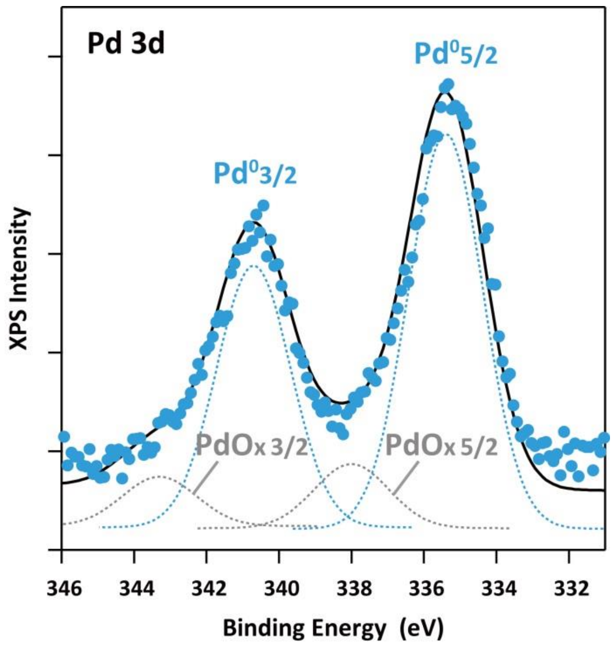

Figure S5 Pd 3*d* XPS profile of *Spirulina*, LH-template 2, after the activation in the biotemplating process; blue dot, measured intensity: black solid line, Gaussian curve given by multi-peak fitting; dotted line, individual peaks identified as Pd metal and PdO_x in the $3d_{5/2}$ and $3d_{3/2}$ ranges.

Table S1 Quantitative analyses of Pd content adsorbed into the *Spirulina* template.

Samples	Pd treatment (min)	ICP results: Pd content per one *Spirulina* (g)	(mol)	(m^3)	(vol %)*1
**LH-template 2**	1	0.4 x 10^{-9}	3.3 x 10^{-12}	0.03 x 10^{-15}	0.5
	3	0.8 x 10^{-9}	7.3 x 10^{-12}	0.06 x 10^{-15}	1.1
	5	1.2 x 10^{-9}	12 x 10^{-12}	0.1 x 10^{-15}	1.7

*1: The **LH-template 2** has an average volume of one *Spirulina* as 6.0 x 10^{-15} m^3, so that the volume fractions of Pd content in vol% were obtained by the ICP-detected Pd volume divided by the volume of *Spirulina* template.

The inductively coupled plasma-optical emission spectrometry (ICP-OES, Shimadzu, ICPS-8100) revealed that the amount of Pd content in the *Spirulina* template was linearly increased as the Pd catalyzation step proceeded up to 5 min (Table S1 and Fig. S6A). The adsorption rate was found as 2.4 x 10^{-12} mol/min given by the slope of fitted line. The Pd content was finally adsorbed to the *Spirulina* template with 12 x 10^{-12} mol for one strain of *Spirulina*, which equals almost 2 vol % in volume fraction of Pd content per one *Spirulina*. It

was also found in observation of energy dispersive X-ray spectrometry (EDX, Oxford Instruments, Swift ED 3000) equipped with SEM (Hitachi, TM3000) that the Pd-based catalyst nuclei formed nanoparticles smaller than the resolution limit of SEM and the Pd nanoparticle was uniformly adsorbed on the surface of *Spirulina* template (Fig. S6B).

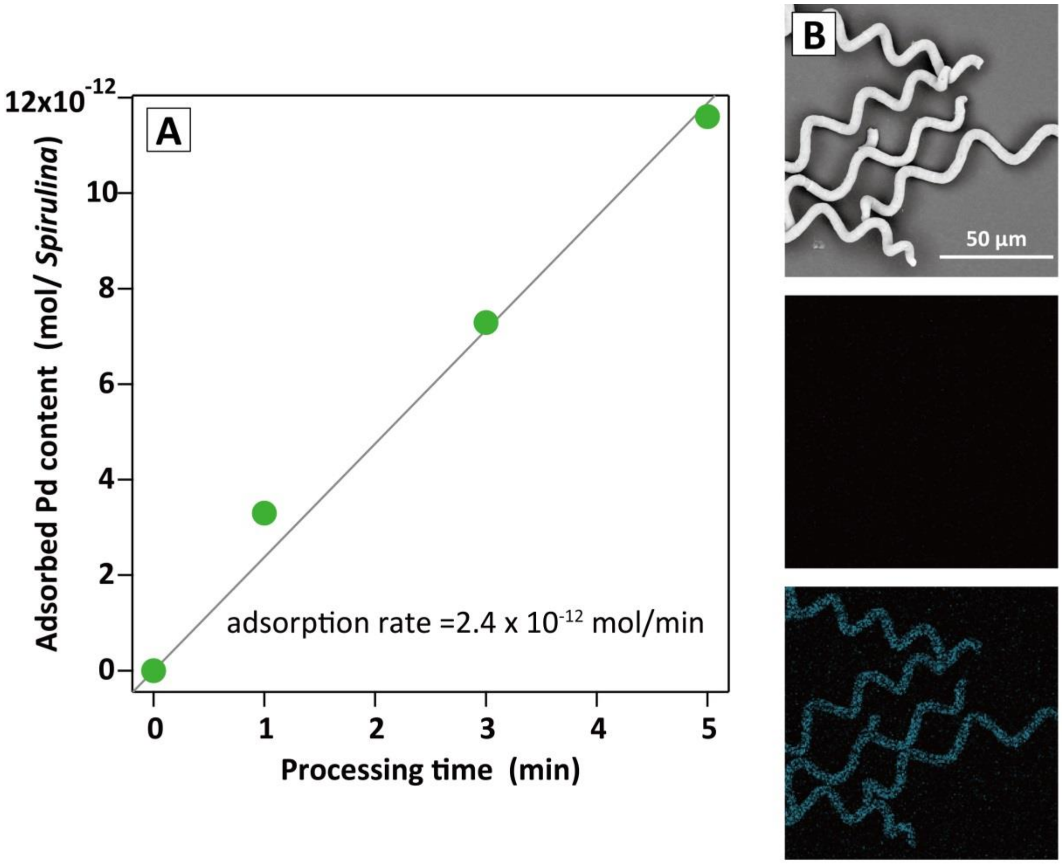

Figure S6 (A) The amount of Pd content per one strain of *Spirulina* (green dot) detected in ICP measurement as a function of treatment time for the Pd catalyzation using OPC-50 Inducer bath. The result was fitted by linear function (solid line) to derive the adsorption rate from the slope. The ICP measurements were carried out with the samples after the activation process (all for 5 min) using OPC-150 Cryster MU. (B) The EDX-SEM observation of *Spirulina* treated with 5-min Pd catalyzation and 5-min activation process: top, back-scattered electron image; middle, Cu Kα mapping; bottom, Pd Lα mapping.

The distribution of Pd catalyst nuclei in/on the *Spirulina* template was identified by EDX-SEM observation (Fig. S7). The Pd Lα mapping visualized the side surface as well as the cross-section formed in intentionally damaged for the observation of Pd distribution inside the

Spirulina. The line analysis was conducted across the cross-section, indicating that the intensity of Pd Lα became higher at the interface of *Spirulina* and kept constant towards a center of the cross-section. The Cu Kα intensity was weaker than the detection limit as a matter of course. It was found here that the Pd ion can be defused into the *Spirulina* tissue and the resulting Pd nanoparticle generated at the stage of activation are equally distributed to the surface and inside of *Spirulina*.

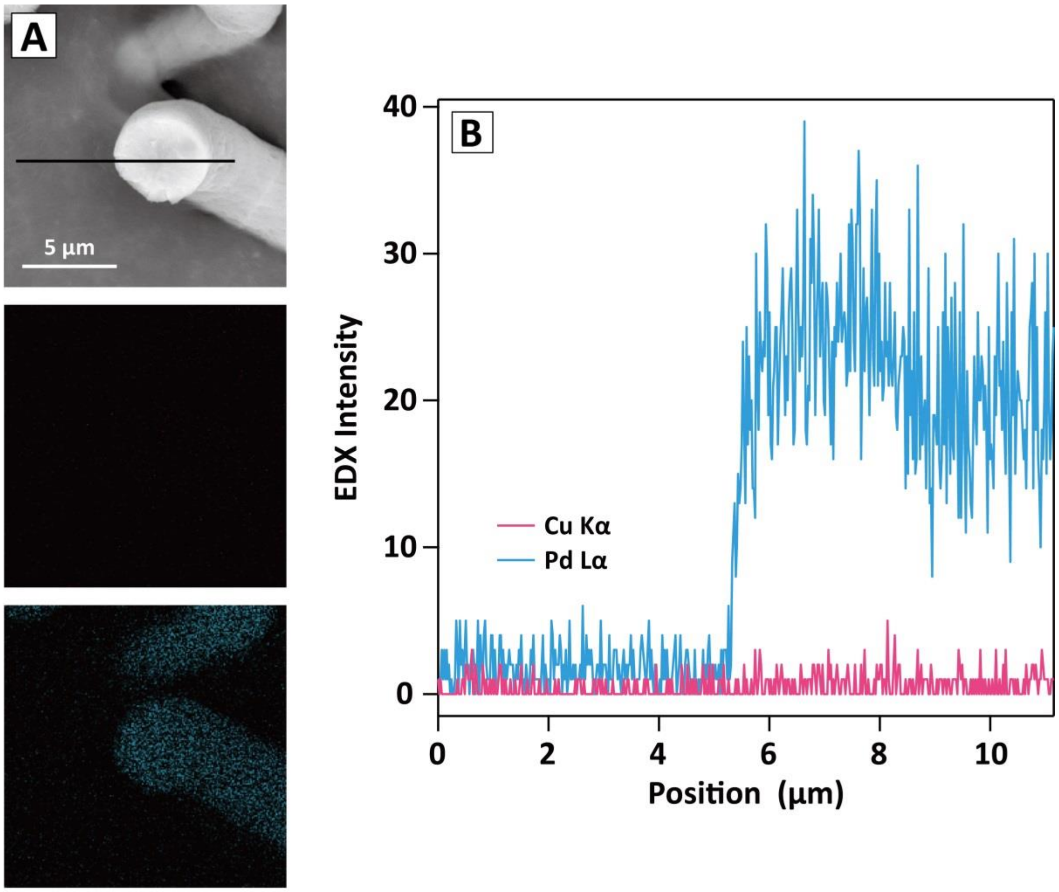

Figure S7 (A) The EDX-SEM observation with back-scattered electron image (top), Cu Kα (middle), and Pd Lα (bottom) mappings. (B) The line analyses for Cu Kα (pink line) and Pd Lα (blue line) across the line indicated in top image of (A), recorded from left to right. The cross-section of *Spirulina* after the activation was intentionally prepared by collapsing the sample on SEM stage with edge of spatula.

In order to confirm the distribution of Pd catalyst nuclei in the *Spirulina*, TEM observation (Jeol, JEM-2100) was employed by using the sample treated up to the activation step (Fig. S8). The sample was microtomed to the direction of long-axis of *Spirulina* without any staining.

The resulting thin-section was found as having contrast high enough to be visualized, indicating uniform distribution of Pd catalyst nuclei as a heavy metal into the *Spirulina*. It is noted that the cell wall as well as the outer membrane can be clearly observed without the staining.

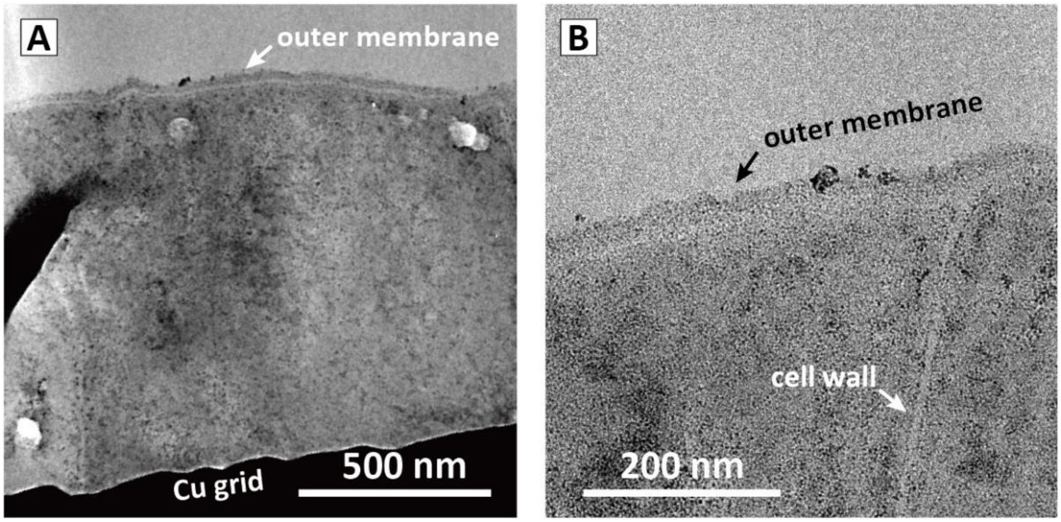

Figure S8 TEM images of thin-section of *Spirulina*. The image of (A) was obtained to show one cell of *Spirulina*, while the (B) was magnified image. The sample after the activation process was used and the staining was not carried out. The thin-section was prepared by microtoming to the direction of long-axis of *Spirulina*.

Copper electroless plating and characterization of product.

The Cu electroless plating was carried out onto the surface of *Spirulina* including the Pd catalyst nuclei as shown in Fig. S9. The Pd nanoparticles adsorbed on the surface can be the plating catalyst, since as the anodic reaction the Pd catalyst oxidizes the reducing reagent in OIC Copper plating bath to produce an electron under alkaline pH. The electron produced from the reducing reagent is consumed to reduce the Cu ion as cathodic reaction, leading to the formation of metallic Cu on the *Spirulina* surface. The resulting Cu on the outermost surface can work as self-catalyst to grow the Cu deposition layer. The XPS revealed that the Cu layer was consisting of metallic Cu (and/or cuprous oxide, Cu_2O, closely overlapped) and a little amount of cupric oxide, CuO (Fig. S10). The experimental curve in the Cu 2*p* region was fitted with Gaussian function at the already-identified Cu $2p_{3/2}$ and Cu $2p_{1/2}$ peak positions[2]. The Cu 2*p* doublet appeared at 932.27 and 952.05 eV was reasonably assigned to metallic Cu and/or Cu_2O with the expected intensity ratio of 2.0 and the other doublet at 934.44 and 953.84 eV was assigned to the CuO. The intensity ratio of peaks in the Cu $2p_{3/2}$ range was 8.7, leading to that 90 % of Cu content was metallic or Cu_2O. The X-ray powder diffraction (XRD, Rigaku RINT-Ultima) pattern gave two major peaks appeared at 43.3 and 50.4 degrees in 2 theta,

consistent with FCC-structured crystalline Cu (Fig. S11). The calculated lattice constants of the Cu μcoil are in good agreement with the standard literature values (JCPDS file No. 04-0836). The peak area of Cu metal was almost 25 times wider than that of Cu oxides. From the result, the deposited layer on the surface was mainly consisting of single phase of crystalline Cu metal with a little amount of oxidized Cu like CuO and Cu_2O.

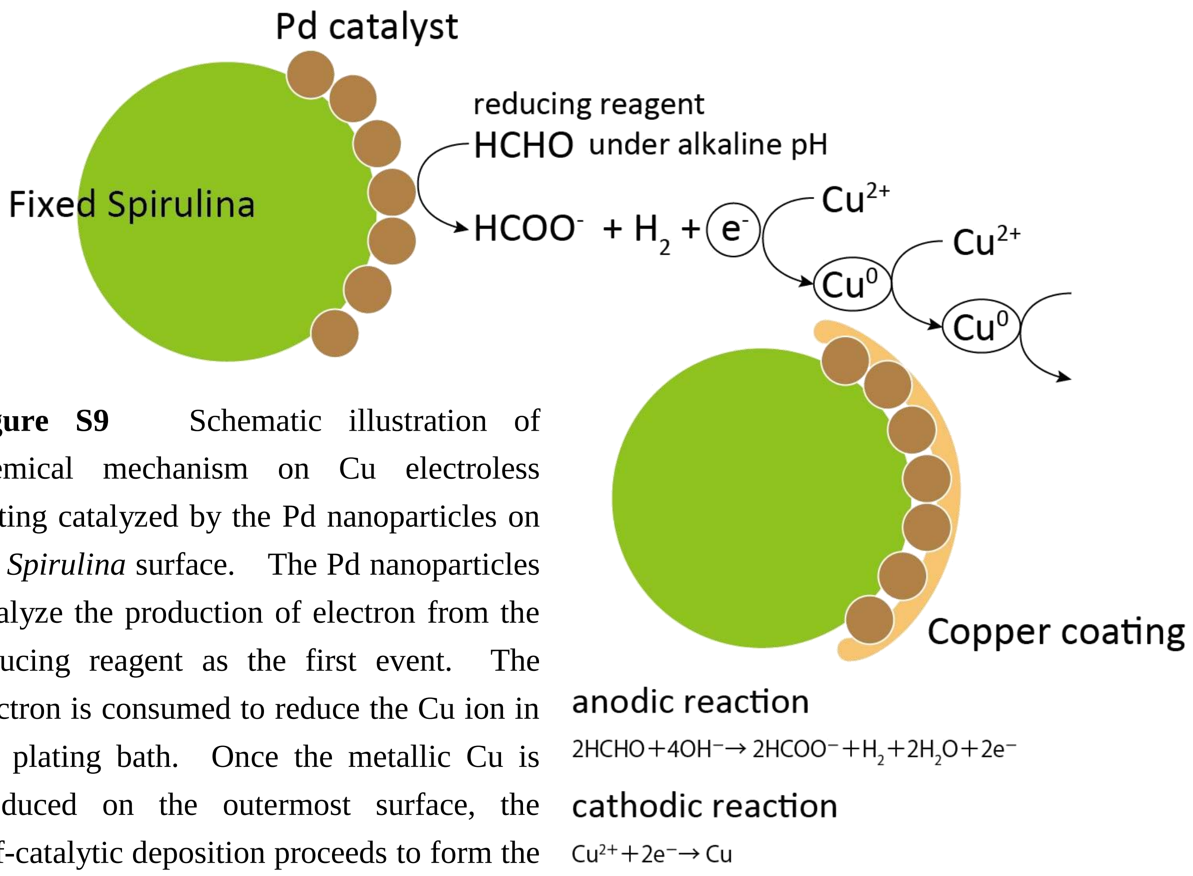

Figure S9 Schematic illustration of chemical mechanism on Cu electroless plating catalyzed by the Pd nanoparticles on the *Spirulina* surface. The Pd nanoparticles catalyze the production of electron from the reducing reagent as the first event. The electron is consumed to reduce the Cu ion in the plating bath. Once the metallic Cu is produced on the outermost surface, the self-catalytic deposition proceeds to form the Cu layer on the *Spirulina* surface.

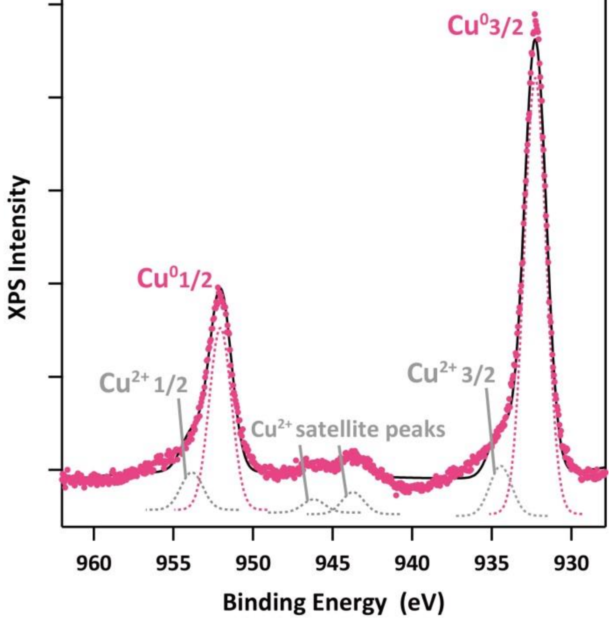

Figure S10 Cu 2*p* XPS profile of Cu μcoil, obtained through the electroless plating for 10 min.; pink dot, measured intensity; black solid line, Gaussian curve given by multi-peak fitting; dotted line, individual peaks identified as Cu metal (and/or closely overlapped Cu_2O) and CuO in the $2p_{3/2}$ and $2p_{1/2}$ ranges.

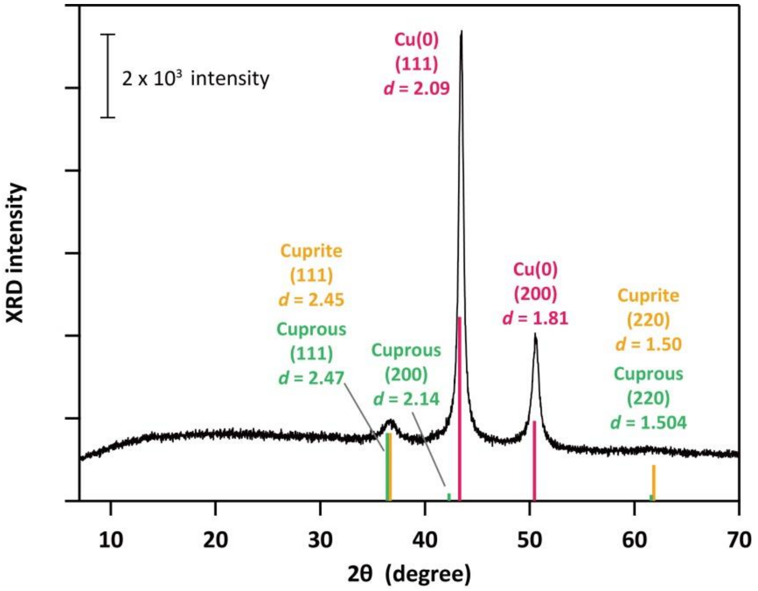

Figure S11 XRD pattern of Cu μcoil with the theoretical peak positions (sticks to zero), *d*-spacings, and crystalline phases of Cu metal and Cu oxides.

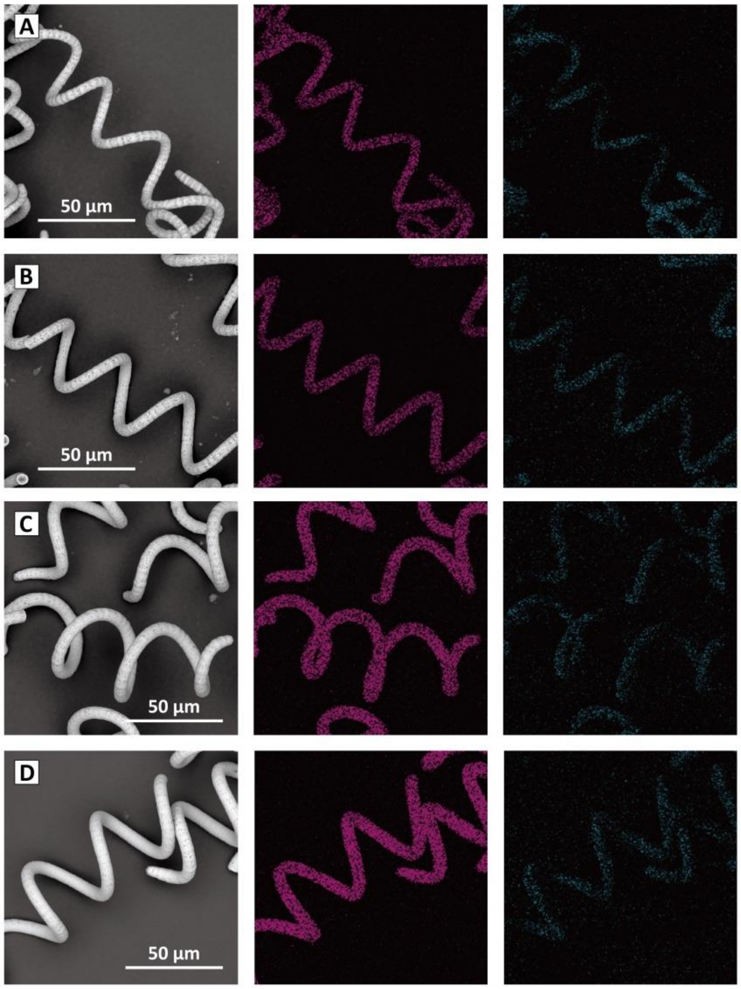

Figure S12 EDX-SEM observation of Cu μcoils obtained through the electroless plating up to 10 min.: left, back-scattered electron image; middle, Cu Kα mapping; right, Pd Lα mapping. The images were taken with the samples at different stage of electroless plating: (A), 2.5 min; (B), 5.0 min; (C), 7.5 min; (D), 10 min.

The EDX-SEM observations were examined to characterize the surface during the electroless plating (Fig. S12). The samples were collected from the plating bath at the different time, *i.e.*, 2.5, 5.0, 7.5, and 10 min. The back-scattered electron images showed higher contrast and the Cu Kα mapping visualized the microstructural feature of μcoils more clearly as the plating reaction proceeded. The Pd Lα mapping gave low contrast throughout the plating process. The EDX spectra indicates that intensities of peaks at 0.93 and 8.04 keV, corresponding to Cu Lα and Kα lines, respectively, increased as a function of plating time (Fig. S13), while an area of peak at 2.83 keV, assigned to Pd Lα, was relatively smaller.

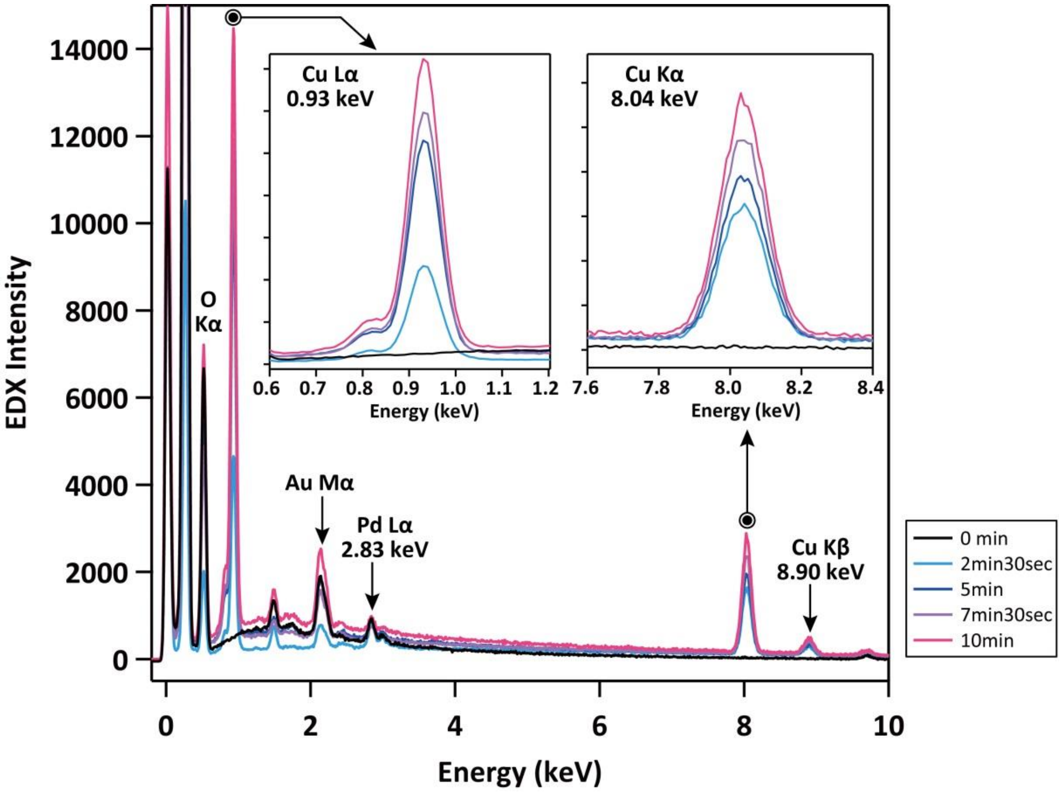

Figure S13 EDX spectra of Copper μcoils prepared at the different plating time. The sample was treated up to the activation process as described in Table S1and successively the electroless plating was conducted for prescribed time. The spectrum at 0 min was obtained from the sample just after the activation, so that only Pd nanoparticles were included in the sample. All the spectra were raw data without offset and normalization. The Au peak was detected because the Au spattering was used to avoid charge-up during the measurements.

SI-IV. Control of Copper layer thickness

In the electroless plating techniques, it has been known that there are several methods to control the thickness of deposited metal layer such as temperature or pH condition. But the loading amount of *Spirulina* suspension is most effective and easily varied in our study. The total surface area of sample against the volume of plating bath, namely, bath load, is essential for the thickness and continuous coating. In order to evaluate the appropriate loading amount for our system, efficiency of Cu deposition was obtained as follows. The efficiency was defined as the volume (m^3) of deposited Cu content to the surface area (S in m^2) of *Spirulina*, *i.e.*, average Cu layer thickness specific in the bath load. The surface are of one *Spirulina* can be derived by $S = \pi d L_{wire} + 2\pi(d/2)^2$. A 40 mL of the *Spirulina* suspension, totally including 4 x 10^6 in the number of *Spirulina*, gives the bath load of 400 cm^2/L, because the surface area of one *Spirulina* is typically in the order of 10^{-4} cm^2. Under this bath load, the Cu electroless plating

was examined and the samples were collected at the different plating time. The ICP analyses revealed that the Cu content increased to 0.31 x 10^{-9} mol (2.2 x 10^{-15} m^3 in the volume) per one *Spirulina* up to 10-min plating, while the Pd content was constant during the plating reaction (Table S2). From the ICP results, the deposition speed and efficiency were found as 22 nm/min and 220 nm, respectively. As double-check, the cross-section of Cu μcoil was observed by SEM (Fig. S14). The resulting μcoil had tubular structure with fixed *Spirulina* template inside as inclusion. It can be assumed that the cytoplasmic components after glutaraldehyde tissue fixation were preserved in the electroless plating bath, and the shape of *Spirulina* was effectively templated. The thickness evaluated from the SEM image was plotted as a function of plating time with the ICP results in Fig. S14. The deposition speed was estimated as 19 nm/min, slightly different from the ICP result. But it should be in allowable range, since the unevenness of plating layer and also the inherent size distribution of *Spirulina* template are considered. From the relationship between the bath load and efficiency, the thickness can be roughly predicted at the different bath load, since the thickness is inversely proportional to the bath load. The loading amount of *Spirulina* suspension with 20 mL was employed to be bath load of 200 cm^2/L in this study, leading to the average Cu layer thickness of 550 nm enough to exhibit bulk-like electric property (Fig. S15).

Table S2 Quantitative analyses of Pd and Cu contents adsorbed into/onto the *Spirulina* template during the electroless plating process

**Samples**	**Cu plating time** (min)	**ICP results: Pd content per one *Spirulina*** (g)	(mol)	(m^3)	(vol %)
**LH-μcoil-2**	2.5	0.59 x 10^{-9}	5.5 x 10^{-12}	0.049 x 10^{-15}	0.8
	5.0	0.55 x 10^{-9}	5.2 x 10^{-12}	0.046 x 10^{-15}	0.8
	7.5	0.62 x 10^{-9}	5.8 x 10^{-12}	0.051 x 10^{-15}	0.9
	10	0.62 x 10^{-9}	5.8 x 10^{-12}	0.052 x 10^{-15}	0.9

**Samples**	**Cu plating time** (min)	**ICP results: Cu content per one *Spirulina*** (g)	(mol)	(m^3)	Deposition efficiency (nm) *1
**LH-μcoil-2**	2.5	7.1 x 10^{-9}	0.11 x 10^{-9}	0.8 x 10^{-15}	80
	5.0	10 x 10^{-9}	0.16 x 10^{-9}	1.2 x 10^{-15}	120
	7.5	16 x 10^{-9}	0.26 x 10^{-9}	1.8 x 10^{-15}	180
	10	20 x 10^{-9}	0.31 x 10^{-9}	2.2 x 10^{-15}	220

*1: The deposition efficiencies are comparable to thickness of Cu layer specific to bath load. The ICP-detected Cu volume per one *Spirulina* template was divided by 1.0 x 10^{-8} μm^2 in the typical average surface area.

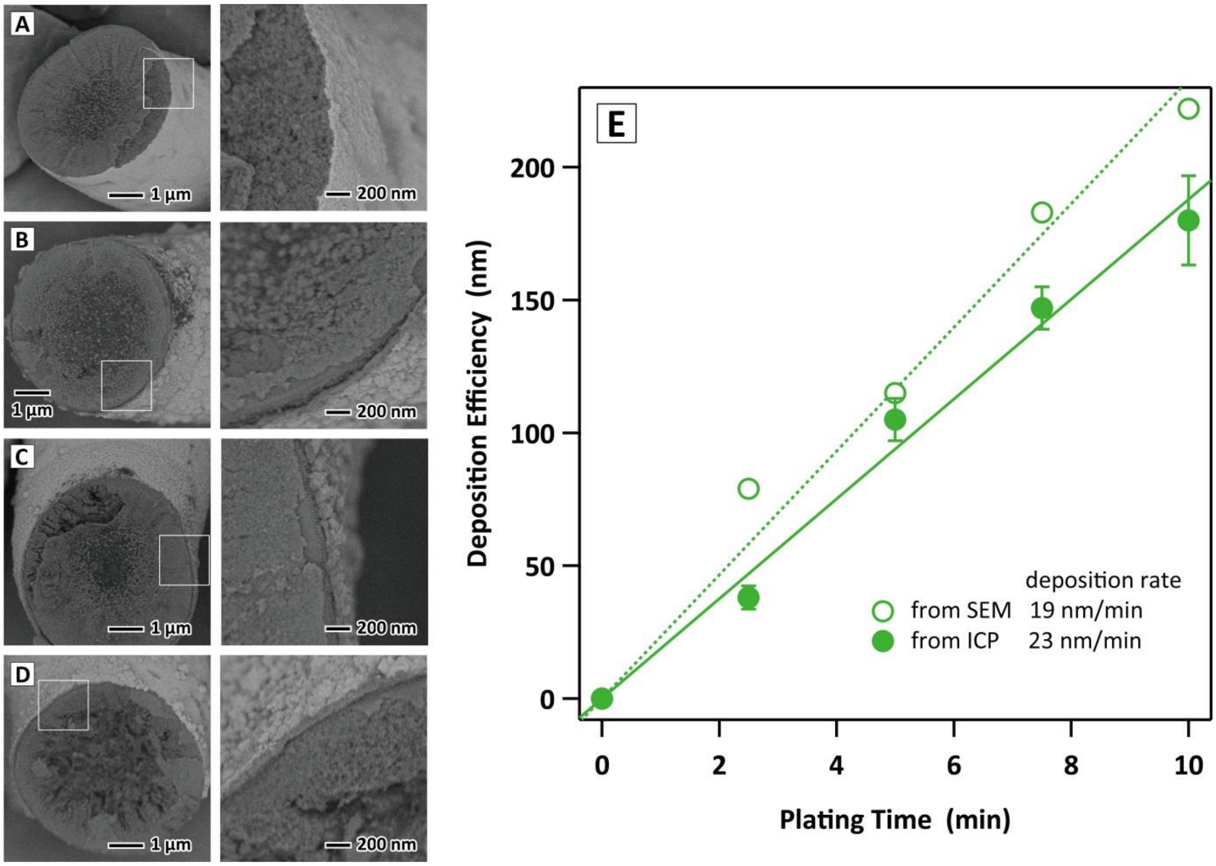

Figure S14 The evaluation of Cu deposition efficiency in the biotemplating process. (A)-(D) SEM images at the cross-sections intentionally produced by collapsing the samples on SEM stages with edge of spatula. The samples were prepared by the plating for (A) 2.5 min, (B) 5.0 min, (C) 7.5 min, and (D) 10 min in the plating time. The right images are magnified at the square areas indicated in the left images. For the detailed measurement of thickness, high-angle back-scattered electron (HA-BSE) mode was used here. (E) The deposition efficiencies are plotted against the plating time in both of the SEM and ICP results.

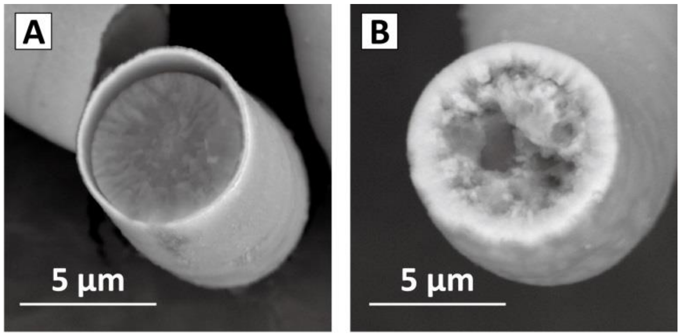

Figure S15 SEM images of Cu µcoils prepared with (A) 400 cm^2/L and (B) 200 cm^2/L in the bath load. The cross-sections were generated by the similar manner to the Fig. S14. The average thickness was changed in the different bath load: (A), 220 nm; (B), 550 nm.

SI-V. Structural parameters of Copper μcoils.

The structural parameters of μcoils were analyzed with the histogram representations (Fig. S16 and S17). The wire diameters (d) were reasonably increased because of the Cu layer formation with electroless plating. The D and L_{free}/N were sufficiently maintained through the biotemplating process. The L_{free}s became shorter with wide relative standard deviations, which may be attributed to brittle damage of the μcoil happened by stirring for the electroless plating.

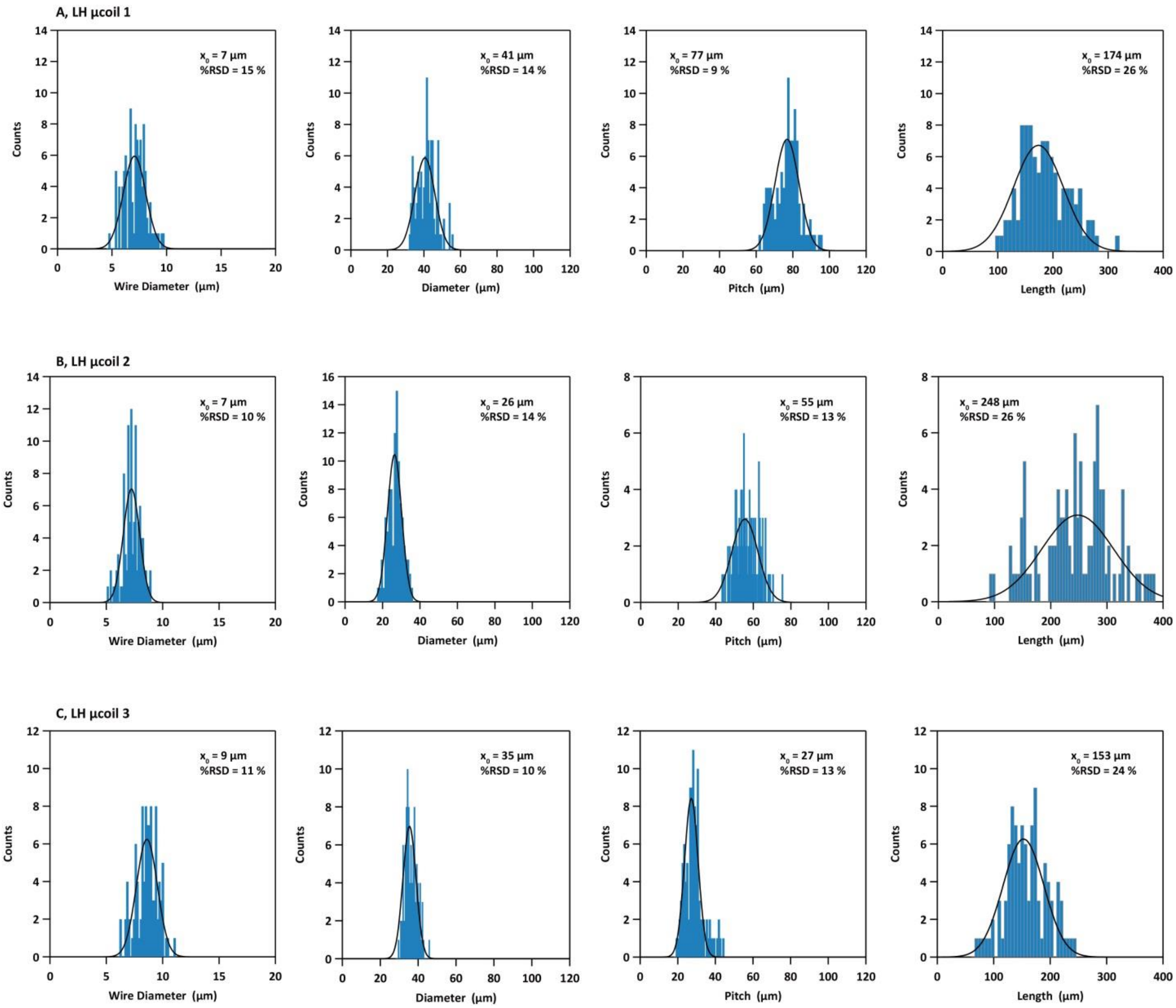

Continued to next page

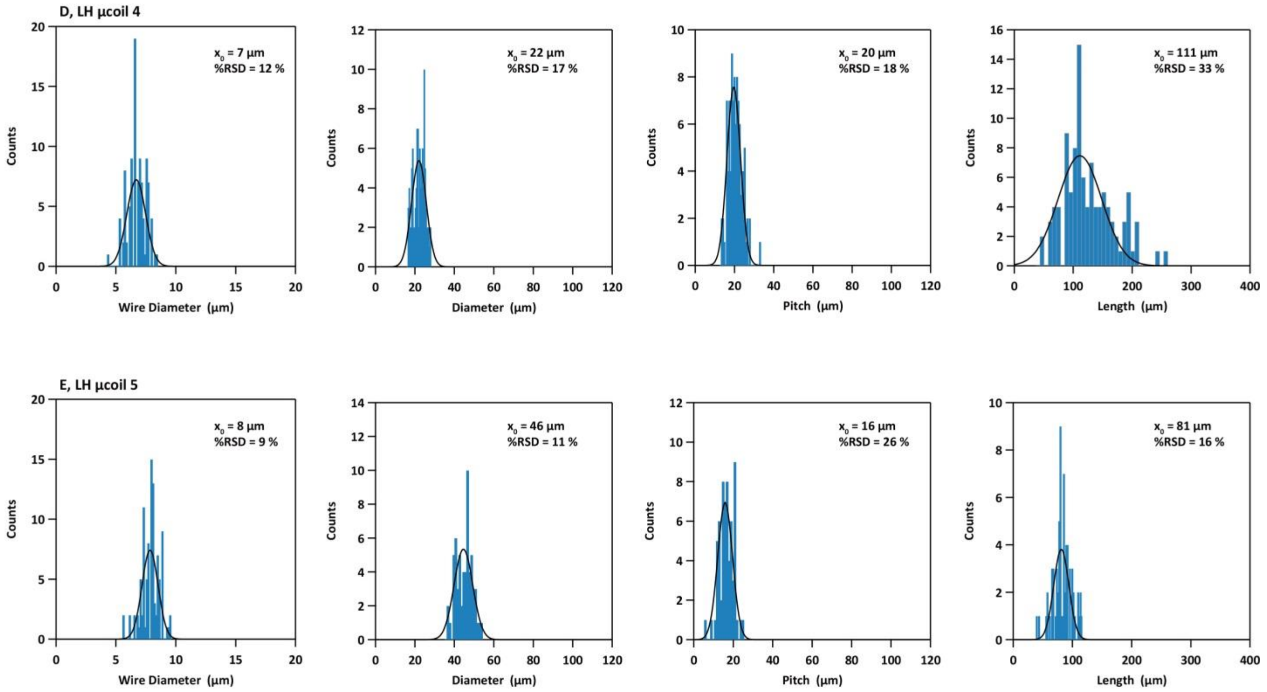

Figure S16 Histograms of structural parameters, wire diameter (*d*), coil diameter (*D*), coil pitch (L_{free}/N), and free length of coil (L_{free}), on helix geometries of LH μcoil-1 to -5. The black lines indicate Gaussian distributions to have averaged values. The average feature size, x_0, and %RSD are indicated in each inset.

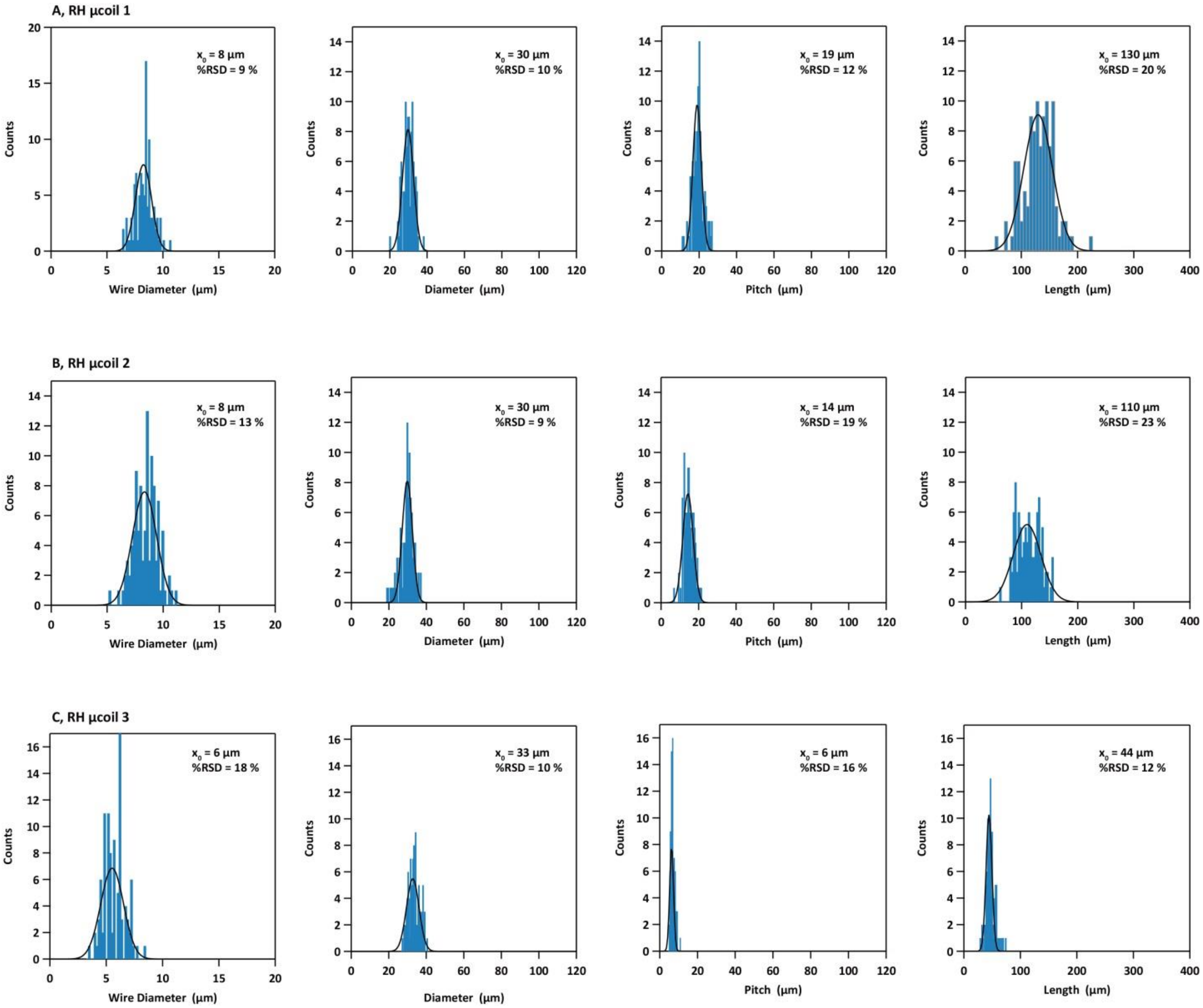

Figure S17 Histograms of structural parameters on helix geometries of RH µcoil-1 to -3.

SI-VI. μcoil-dispersed sheet for evaluation of electromagnetic response

The electromagnetic property within GHz range was analyzed at Japan Fine Ceramics Center. Free space method (S-parameter method) was employed for transmittance and reflectance in the every range of Fig. 4a and 4b; K-band, 18-26.5 GHz; Ka-band, 26.5-40 GHz; V-band, 50-75 GHz; W-band, 75-110 GHz.

We prepared μcoil-dispersed sheets by using paraffin (mp 70-80 °C, Sigma-Aldrich) as a transparent matrix. A prescribed amount of μcoils was dispersed into paraffin melt at 90 °C. The uniformly dispersed paraffin melt was solidified in a mold, affording a μcoil-dispersed paraffin sheet with 4 cm square and 1 mm thickness (Fig. S18). A 1 wt % of the μcoil in the paraffin sheet corresponds to from 10^{11} to 10^{12} coils m^{-3} in number density. The above procedure allowed us to suppress <10 % of entangled μcoils as so-called lump, which causes undesirable contributions in the measurements. The paraffin-based sheets were used for the K- and Ka-bands, and THz wave region. For the V- and W-bands, silicon matrix was used for the fabrication of sheet with 30 cm square and 1 cm thickness.

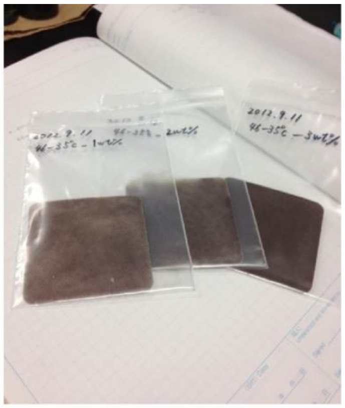

Figure S18 The photo of μcoil-dispersed paraffin sheets.

The optical densities of μcoil-dispersed paraffin sheets were converted from the transmittances shown in Fig. 4C of the main text (Fig. S19). The intensities at 1.0 THz were plotted against the number densities of μcoils in the paraffin sheets. Since the linear relationship was reproduced with its slope of *ca*. 1.5×10^{-12} with less than 4 % of relative standard deviation, the dispersibility of μcoils into paraffin was guaranteed.

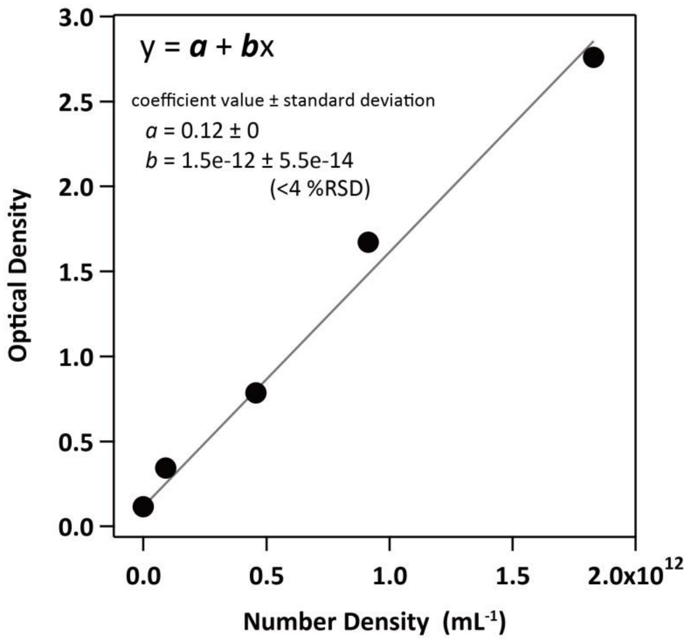

Figure S19 Optical density of LH μcoil-1 at 1 THz as a function of the number density in the paraffin sheet. The optical densities were obtained by conversion from the transmittances shown in Fig. 4C of main text. The solid line indicates line approximation with small error (<4 %RSD).

The normal mode transmission spectra (non-polarization mode) corresponding to Figure 5a were summarized in Figure S20. Every sample shows low transmittance less than 10 %, while the loose LH μcoil-1 is only significant in regard to very low transmittance reaching to 0.01%.

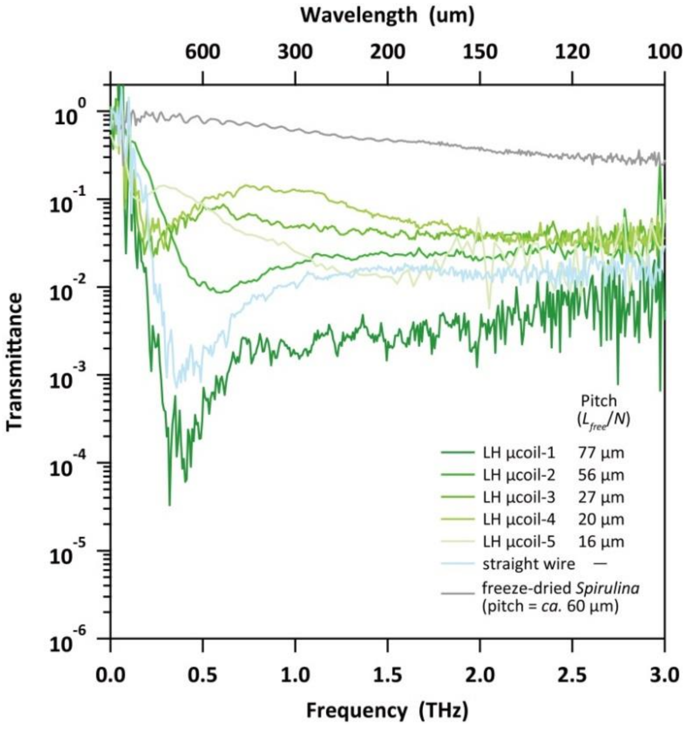

Figure S20 Transmission spectra of series of LH μcoil under non-polarization mode. The sample concentration was 2 wt% in every cases.

SI-VII. THz-TDS-PA setup

A transmission-type THz-TDS system combined with polarimetric analysis was conducted to evaluate the electromagnetic response of Cu μcoils. Femtosecond laser pulses from mode-locked Ti:sapphire laser with pulse width of 100 fs, center wavelength of 800 nm, and time average power of 5.0 mW irradiated gaps of photoconductive dipole antenna fabricated on low-temperature-grown GaAs substrate. The emitter dipole antenna was a.c. biased at 40 V and 3 kHz to generate THz pulses ranging from 0.2 THz to 3.0 THz. The emitted THz wave was collimated by an off-axis paraboloidal reflector to propagate through the sample toward a second paraboloidal reflector. The focused THz wave reached to the detector dipole antenna, which was triggered by gate pulse separated from the laser pulse through optical time delay stage. For the polarization analysis, three wire grid polarizers (WG1 to WG3) were inserted: WG1, between the emitter and the sample to linearly polarize the THz wave; WG2, between the sample and WG3 with +45 ° or -45 ° against the horizontal direction to analyze the polarization of THz wave transmitted through the sample; WG3, between WG2 and the detector to extract the polarization component parallel to the most sensitive direction for the detector. By scanning the time delay stage, the time evolution of the polarization state of THz wave transmitted from the samples were obtained. Transmission intensities of left- and right- handed circular polarizations (LCP, RCP) with their phase shifts are obtained from the x- and y-axis components (E_x and E_y) of the transmitted THz waveforms in the time domain. The Fourier transformation applied to the transmitted THz wave derived the transmission spectra, ellipticity, γ, and rotation angle, η, in the frequency domain.

In order to define the sense of rotation of emitted elliptical polarization, it should be noted that the transmitted wave was observed *against* the direction of propagation from the detector, *i.e.*, the complex amplitude of the RCP and LCP of the THz waves, E_R and E_L are obtained as $E_R = \frac{1}{\sqrt{2}}(E_x - iE_y)$ and $E_L = \frac{1}{\sqrt{2}}(E_x + iE_y)$, respectively. Here, the phase of the THz wave is defined by $\theta = \omega t - kz + \phi$, where ω is angular frequency, k is wave vector, and ϕ is phase. The polarization state is described with $\tan\gamma = \frac{|E_R|-|E_L|}{|E_L|+|E_R|}$ and $\eta = \frac{1}{2}\arg\frac{E_L}{E_R}$.

As became clear above, the spectrum obtained from THz-TDS under non-polarization mode should trace an average of those against LCP and RCP in polarization mode, THz-TDS-PA.

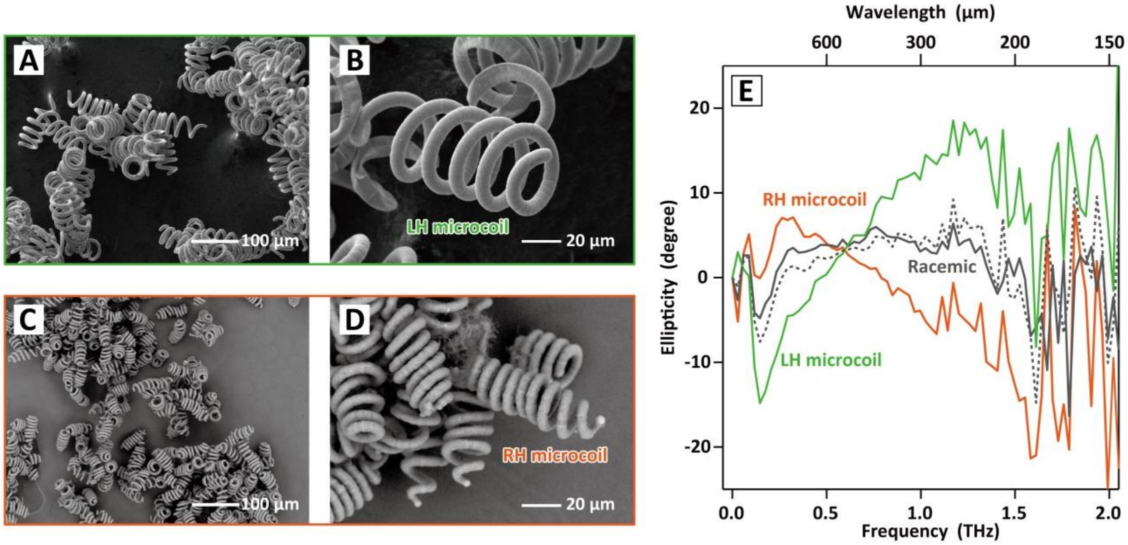

Figure S21 Dependence of µcoil handedness on ellipticity spectra. SEM images of (**A**), (**B**) LH µcoil-5 and (**C**), (**D**) RH µcoil-2. (**E**) Ellipticity spectra of LH (green line), RH (orange line) µcoils, and racemic mixture (gray solid line) with 2 wt% of concentration for all the samples. The average spectrum of ellipticities of LH and RH µcoils is also added in dotted gray line. The LH and RH µcoils afforded nearly mirror-image ellipticity spectra over the full spectral range, while the racemic mixture showed almost flat spectral feature.

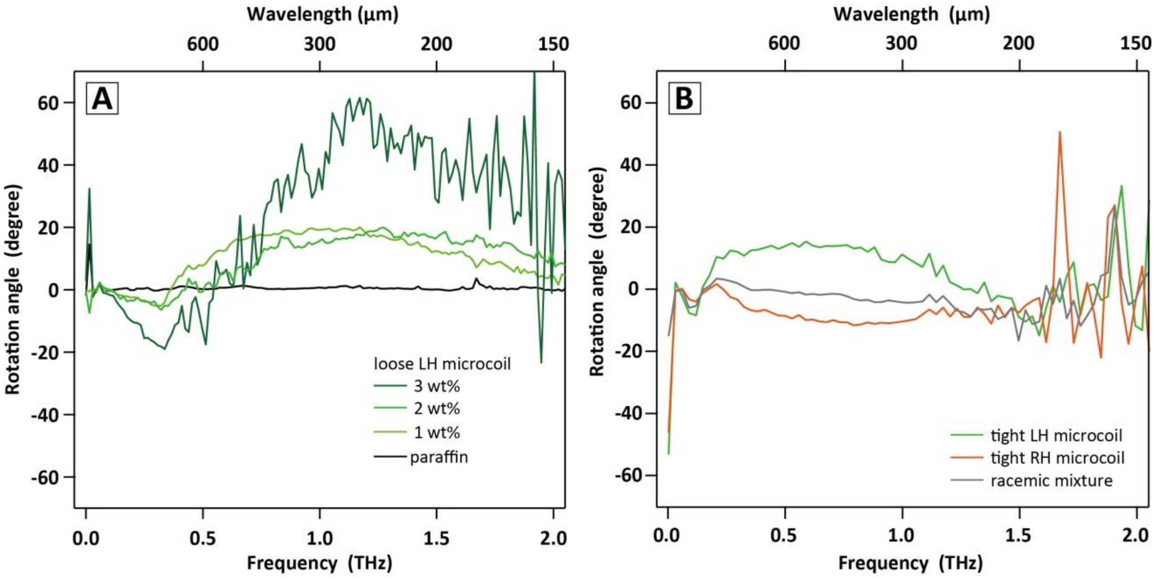

Figure S22 Rotation angle spectra of µcoil sheets including (A) loose LH µcoil-2 with different weight concentrations and (B) the enantiomeric pair of LH µcoil-5 and RH µcoil-2 (2 wt%).

The handedness of μcoil was firmly identified by SEM observation (Fig. S21A to S21D). Correspondent pair of LH and RH μcoils in respect to L_{free}/N and D was selected to examine the optical chirality, *i.e.*, LH μcoil-5 and RH μcoil-2. This enantiomeric pair exhibited ellipticity spectra with opposite sign, while the observable ellipticity disappeared for racemic form intentionally mixed with two of them (Fig. S21E). The racemic mixture resulted in being offset the ellipticity expressed by the handedness of μcoil.

The rotation angle spectra were also obtained for the same series as the above experiment (Fig. S22). It can be confirmed that the LH and RH μcoils showed dextrorotation and laevorotation, respectively. Since the μcoil effectively absorbs circularly-polarized wave rotating to the same direction as the handedness of μcoil, the oppositely-rotating circular polarization only can transmit through the μcoil-dispersed sheet.

SI-VIII. Geometric parameters of samples

Table S3 The geometric parameters of LH μcoils and predictable operation frequencies

Parameters	symbol	Units	LH μcoil-1	LH μcoil-2	LH μcoil-3	LH μcoil-4	LH μcoil-5
wire diameter	d	μm	7	7	9	7	8
coil diameter	D	μm	41	26	35	22	46
free length of pitch	L_{free}/N	μm	77	56	27	20	16
number of turn	N	-	2.3	4.5	5.7	5.6	5.1
free length of coil	L_{free}	μm	174	248	153	111	81
length of wire for one pitch	L_{wire}/N	μm	150	99	113	72	145
length of wire for one coil	L_{wire}	μm	339	441	642	399	736
cross-sectional area of Cu layer[1]		m^2	13×10^{-12}	13×10^{-12}	16×10^{-12}	13×10^{-12}	15×10^{-12}
volume of one coil[2]	V_{coil}	m^3	4.4×10^{-15}	5.8×10^{-15}	11×10^{-15}	5.2×10^{-15}	11×10^{-15}
mass of one coil[3]	M	g/(one coil)	40×10^{-9}	52×10^{-9}	95×10^{-9}	47×10^{-9}	97×10^{-9}
pitch angle[4]	α	degree	30.9	34.0	13.8	16.1	6.3
theoretical operation frequency (pitch)[5]	F_d	THz	0.67-1.33	1.02-2.03	0.88-1.77	1.39-2.78	0.69-1.38
(total length)		THz	0.29-0.59	0.23-0.45	0.16-0.31	0.25-0.50	0.14-0.27
experimental frequency range[6]		THz	0.5-1.5	0.5-1.6	0.5-1.9	0.5-2.1	0.4-1.2

[1]The cross-section is ring-shape consisting of Cu layer with 550 nm thickness. [2]The V_{coil} only includes volume of Cu shaping one coil. [3]The M is obtained by multiplying V_{coil} by theoretical density of Cu metal, 8.94×10^6 g/m^3. [4]The pitch angle equals to $\tan^{-1}(L_{free}/N) / (\pi D)$. [5]The region of frequency means that the μcoil emits elliptical polarization with the opposite handedness within the range and can be predicted with $L_{wire}/N < \lambda_0 < 2L_{wire}/N$, as defined in helical antenna array. The wave propagates in paraffin matrix (n = 1.5), so that the operation frequency is given by $F_d = \frac{300 \times n}{\lambda_0}$. [6]The frequency region was defined as the difference between two peaks of ellipticity angles.

Table S4 The geometric parameters of RH µcoils and predictable operation frequencies

Parameters	symbol	Units	RH µcoil-1	RH µcoil-2	RH µcoil-3	Straight wire[1]	Freeze-dried *Spirulina*[2]
wire diameter	d	µm	8	8	6	6	5
coil diameter	D	µm	30	30	33	*n/a*	20
free length of pitch	L_{free}/N	µm	19	14	6	*n/a*	44
number of turn	N	—	6.8	7.9	7.0	*n/a*	4.3
free length of coil	L_{free}	µm	130	110	44	320	188
length of wire for one pitch	L_{wire}/N	µm	96	95	104	*n/a*	77
length of wire for one coil	L_{wire}	µm	658	749	725	320	328
cross-sectional area of Cu layer		m^2	15×10^{-12}	15×10^{-12}	11×10^{-12}	11×10^{-12}	*n/a*
volume of one coil	V_{coil}	m^3	9.7×10^{-15}	11×10^{-15}	8.2×10^{-15}	3.6×10^{-15}	*n/a*
mass of one coil	M	g/(one coil)	87×10^{-9}	99×10^{-9}	73×10^{-9}	32×10^{-9}	*n/a*
pitch angle	α	degree	11.4	8.5	3.5	90	35
operation frequency in paraffin (pitch)	F_d	THz	1.04-2.08	1.05-2.10	0.96-1.93	*n/a*	1.30-2.61
(total length)		THz	0.15-0.30	0.13-0.27	0.14-0.28	0.31-0.63	0.31-0.61
experimental frequency range		THz	0.4-1.6	0.3-2.0	*n/a*	*n/a*	*n/a*

[1]The sample formed straight shape, so that V_{coil} and M were described as the volume and mass of Cu wire, respectively. [2]The F_d values were simply obtained from the sample shape regardless of the presence or absence of Cu metal coating (electroconductivity). The *n/a* indicates the parameter inapplicable to the sample.

1 Fiermans, L., Gryse, R. D., Doncker, G. D., Jacobs, P. A. & Martens, J. A. Pd Segregation to the Surface of Bimetallic Pt-Pd Particles Supported on H-β Zeolite Evidenced with X-Ray Photoelectron Spectroscopy and Argon Cation Bombardment. *J. Cat.* **193**, 108-114 (2000).

2 Ghijesen, J. et al. Electronic structrue of Cu_2O and CuO. *Phys. Rev. B* **38**, 11322-11330 (1988).
